# Metabolite Profiles in Response to Dietary Interventions for Management of Blood Pressure: A Systematic Review

**DOI:** 10.1007/s13668-025-00676-7

**Published:** 2025-06-20

**Authors:** María Gómez-Martín, Floor Rikken, Erin D. Clarke, Jordan Stanford, Jessica J. A. Ferguson, Clare E. Collins

**Affiliations:** 1 School of Health Sciences, College of Health, Medicine and Wellbeing, The University of Newcastle, Callaghan, NSW 2308 Australia; 2 Food and Nutrition Research Program, Hunter Medical Research Institute, New Lambton Heights, NSW 2305 Australia; 3 Division of Human Nutrition and Health, Wageningen University and Research, Wageningen, 6702 PB The Netherlands

**Keywords:** Diet, Metabolites, Blood pressure, Hypertension, Systematic review

## Abstract

**Background:**

Hypertension is a major global public health issue. The mechanisms through which diet influences blood pressure (BP) remain to be fully elucidated. Nutritional metabolomics offers an objective method for examining diet-health outcomes, including the contribution of intermediate molecules and metabolic byproducts or metabolites to BP regulation. To date, no review has investigated the relationship between diet, metabolites and BP regulation.

**Objective:**

This systematic review aim was to synthesise findings of human dietary intervention studies on BP, including feeding studies providing all foods and meals, as well as those that provided supplements.

**Methods:**

Six databases were systematically searched (Scopus, Medline, Embase, CINAHL, PsycINFO, and Cochrane) for intervention studies examining the relationship between dietary metabolites and BP regulation. The Academy of Nutrition and Dietetics Quality Criteria were used to assess the risk of bias.

**Results:**

Twelve articles (11 unique studies) met the inclusion criteria, reporting 11 significant associations between metabolites and BP, while one study found no significant associations. More than 100 metabolites were associated with BP, 40 associated with SBP, 29 with DBP, 31 with both, and 2 did not differentiate between SBP or DBP. Only two metabolites, proline-betaine and N-acetylneuraminate, had significant relationships with BP measurement in more than one study.

**Conclusions:**

This review identified a shortlist of potential metabolite indicators of response to dietary interventions for BP regulation. Findings highlight nutritional metabolomics as a potential contributor to understanding diet-induced changes in BP and CVD risk reduction. However, variability in reported metabolites and limited replication across studies may affect specificity and limit generalizability. Further research is needed to better understand this relationship.

**Supplementary Information:**

The online version contains supplementary material available at 10.1007/s13668-025-00676-7.

## Introduction

Hypertension is a major global public health issue [1, 2]. This physiological condition is defined as elevated blood pressure (BP), with systolic BP (SBP) ≥ 140 mmHg and/or diastolic BP (DBP) ≥ 90 mmHg [1]. Hypertension is a major risk factor for cardiovascular disease (CVD) [3] and in 2019, high SBP was the leading level-two risk factor of the Global Burden of Diseases for mortality, accounting for 10.8 million deaths worldwide, a two-fold increase compared to data from 1990 [1, 3]. Furthermore, hypertension was the main risk factor contributing to disability-adjusted life-years among adults aged ≥ 50 years [3]. Given that reducing BP reduces overall CVD risk, the importance of identifying and managing modifiable factors, such as diet, is underscored [4].

The World Health Organisation recommends a healthy diet as one of the most effective ways to prevent or reduce elevated BP [2]. Several dietary interventions have sought to evaluate the role of diet in BP regulation [5]. The Dietary Approaches to Stop Hypertension (DASH) diet, a diet rich in fruits, vegetables, wholegrains, low-fat dairy products, and low in sodium, saturated and total fat, has been shown to substantially lower BP (reduced SBP and DBP by 5.5 and 3.0 mm Hg more, respectively, than the control diet (*p* < 0.001 for each)) [6]. In addition, a meta-analysis showed that the DASH diet significantly reduced SBP by 6.74 mmHg (95%CI: -8.25, -5.23, I(2) = 78.1%) and DBP by 3.54 mmHg (95%CI: -4.29, -2.79, I(2) = 56.7%) [7]. Similar results have been reported using the Mediterranean Diet, characterised by high intakes of vegetables, fruits, wholegrains, nuts, and extra virgin olive oil [8]. However, while the evidence for the antihypertensive effect of the DASH diet is strong and consistent, findings for the Mediterranean diet have been more variable, with some meta-analyses reporting insufficient evidence and others supporting its effectiveness [9–11]. In addition, a network meta-analysis observed that DASH diet was more effective in reducing SBP compared to Mediterranean (MD: -3.31 mmHg, 95% -6.20, -0.42), suggesting the DASH diet could be the most effective dietary approach to improve BP [9].

The impact of individual micronutrients on change in BP has also been explored. Research shows that higher sodium intake is associated with higher BP, while the opposite has been observed with potassium intake [12–15]. A meta-regression of randomised controlled trials found that increased potassium excretion and a lower sodium-to-potassium ratio were associated with reduced blood pressure (*p* < 0.05) [16]. The Salt Substitute and Stroke Study (SSaSS) (*n* = 20,995, rural China) demonstrated that potassium-enriched salt significantly reduced the risk of stroke (14%, *p* = 0.006), major cardiovascular events (13%, *p* < 0.001), and premature death (12%, *p* < 0.001) over five years [17].

Despite the reported health benefits of specific dietary interventions, individuals’ metabolic responses to diet and food products may vary, and the underlying mechanisms are yet to be fully elucidated [18].

Precision nutrition aims to utilise data from genomics, proteomics and/or metabolomics to assess an individual’s response to consumption of specific foods or dietary patterns [19, 20]. Nutritional metabolomics is an emerging research field that seeks to enhance the precision of assessing and quantifying dietary intakes but also investigates how metabolic responses differ in response to different diets in the context of health and disease [19, 21–23]. Metabolomic profiling has the capability to identify changes in small metabolites in response to modifications in the environment, including diet [23]. By measuring intermediate molecules and by-products generated during metabolism, metabolic profiles can offer an objective approach to assessing individual dietary intakes and variability in metabolic responses [23], which in turn are influenced by genetic factors [24]. The potential to detect changes in metabolites during dietary interventions holds promise in precision nutrition in terms of accounting for inter-individual variation in metabolic response to dietary interventions and evaluation of the relationship with health outcomes [23].

Several papers have identified metabolites associated with BP [25–27]. A recent review highlights that dietary fiber serves as the primary substrate for gut microbiota in the production of short-chain fatty acids, metabolites with beneficial effects on cardiovascular health [28]. This suggests that diet-derived metabolites contribute to the mechanisms underpinning BP regulation, and that further exploration into the potential role they may play in lowering CVD risk is warranted.

Currently, there is no comprehensive review that has synthesised evidence from human clinical trials evaluating the relationship between diet-related metabolites and BP. Therefore, this review aims to synthesise findings from human intervention trials that evaluated the relationship between diet-related metabolites and BP outcomes, in response to provision of food, meals, or dietary supplements, compared to a control intervention in randomised controlled trials.

## Methods

The systematic review followed the Preferred Reporting Items for Systematic Reviews and Meta-Analyses (PRISMA) guidelines with the PRISMA-checklist presented in Table S1 [29]. The review protocol was registered on Open Science Framework (10.17605/OSF.IO/A9BKE).

### Search Strategy

Six major databases were thoroughly searched from inception through to November 12th, 2024 (FR and MG-M). These databases included EMBASE, Medline, CINAHL, PsychINFO, Scopus and Cochrane, using the following search terms (Table S2): “metabolome”[tiab] OR “metabolites“[tiab] OR “metabolomics“[tiab] OR “metabolomics“[Mesh]) AND (“diet“[tiab] OR “diet“[mesh] OR “dietary“[tiab] OR “feeding study“[tiab]) AND (“blood pressure“[tiab] OR “hypertension“[tiab] OR “systolic” [tiab] OR “diastolic” [tiab]) AND (“intervention“[tiab] OR “trial“[tiab]). The limits included full-text publications available in English and studies conducted in humans.

### Eligibility Criteria

The research question was defined using the PICOS (participants, intervention, comparator, outcome, setting) criteria (Table 1). For the current review, dietary interventions studies have been defined as those in which; (1) all foods and/or meals were provided during all or part of the intervention; (2) key foods that define a dietary pattern were provided, along with a dietary pattern prescription; and (3) a single food was provided, regardless of duration. Meanwhile, the following studies were excluded: interventions during pregnancy, prescriptive dietary interventions only, observational and cohort studies, postprandial interventions and studies administering concentrated metabolite supplementation.

**Table 1:** PICOS (participants, intervention, comparator, outcome, setting) inclusion and exclusion criteria

	Inclusion	Exclusion
Participants	• Adults ≥ 18 years • No restrictions on the country of study	• Pregnancy
Intervention	• Dietary interventions (e.g., whole diet) • Only one food product/meal or supplements • Randomised controlled trials • Pre-post studies	• Concentrated metabolite supplementation • Observational studies (i.e. cohort, cross-sectional designs) • Review papers, conference abstracts • Postprandial interventions • Prescriptive dietary interventions only
Comparator	• Control (e.g., usual diet) • Alternative feeding/dietary pattern or food product/meal/placebo	
Outcome	• Metabolites biological samples (e.g., serum, plasma, urine, faeces) and BP (Systolic BP and Diastolic BP)	
Setting	• Free living studies where the dietary intervention is provided • Dietary intervention conducted in a research lab/facility where participants consume whole diet under supervision.	• Laboratory based feeding studies where single foods or meals are fed and acute postprandial responses measured

BP, blood pressure

### Data Selection

The search outputs were saved in Endnote and uploaded into Covidence systematic research platform for screening (Veritas Health Innovation, Melbourne, www.covidence.org). Two independent reviewers (FR, EC, JS, JF, MG-M or CC) screened titles and abstracts of all retrieved papers, constituting a double-screening process. Disagreement was solved by discussion between two researchers and if agreement could not be reached, an independent third party (EC or JS) would make the final decision. Full texts retrieved were assessed for eligibility by two independent reviewers (FR, EC, JS, JF, MG-M or CC), and conflicts resolved by a third independent investigator (FR, EC, JS, JF or MG-M).

### Data Extraction, Synthesis and Quality Assessment

Data extraction was performed by FR and MG-M using a data extraction spreadsheet for study details, including year, location, study design, population characteristics and study characteristics. Data was synthesised narratively and reported overall, by dietary protocol and by biofluid type. In addition, data from blood pressure measurements were added, where available (reported as mean ± standard deviation (SD), mean ± standard error of the mean (SEM) or mean changes from baseline). The methodological quality of the included studies was assessed using the Quality Criteria Checklist in *The Academy of Nutrition and Dietetics*,* Evidence Analysis Manual: Steps in the Academy Evidence Analysis Process* [30] in duplicate by two independent researchers (FR, EC, JS, JF or MG-M). The Quality Criteria Checklist contains 10 structured validity questions including additional sub-questions specific to different study designs. The following criteria appraised the risk of bias and scientific quality: research question, selection of participants, comparability of study groups, withdrawal handling and blind methods (where relevant), description of intervention(s), procedures and intervening factors, validity and reliability of measurements, definition of outcome measures, statistical methods including sample size and power, conclusions, biases and potential limitations considering funding, sponsorships or other conflicts of interest. An overall rating (i.e., ‘positive’, ‘negative’ or ‘neutral’) was assigned to each study. A study was deemed ‘positive’ if it met all priority criteria and most of the validity criteria. A study was given a ‘neutral’ rating if it met most of the validity criteria but did not meet one or more for the priority criteria. A ‘negative’ rating was given to a study that failed to meet six or more of the validity criteria. A third independent researcher assisted in the resolution of quality ratings for studies where there were discrepancies between the two independent researchers.

## Results

**Fig. 1:**
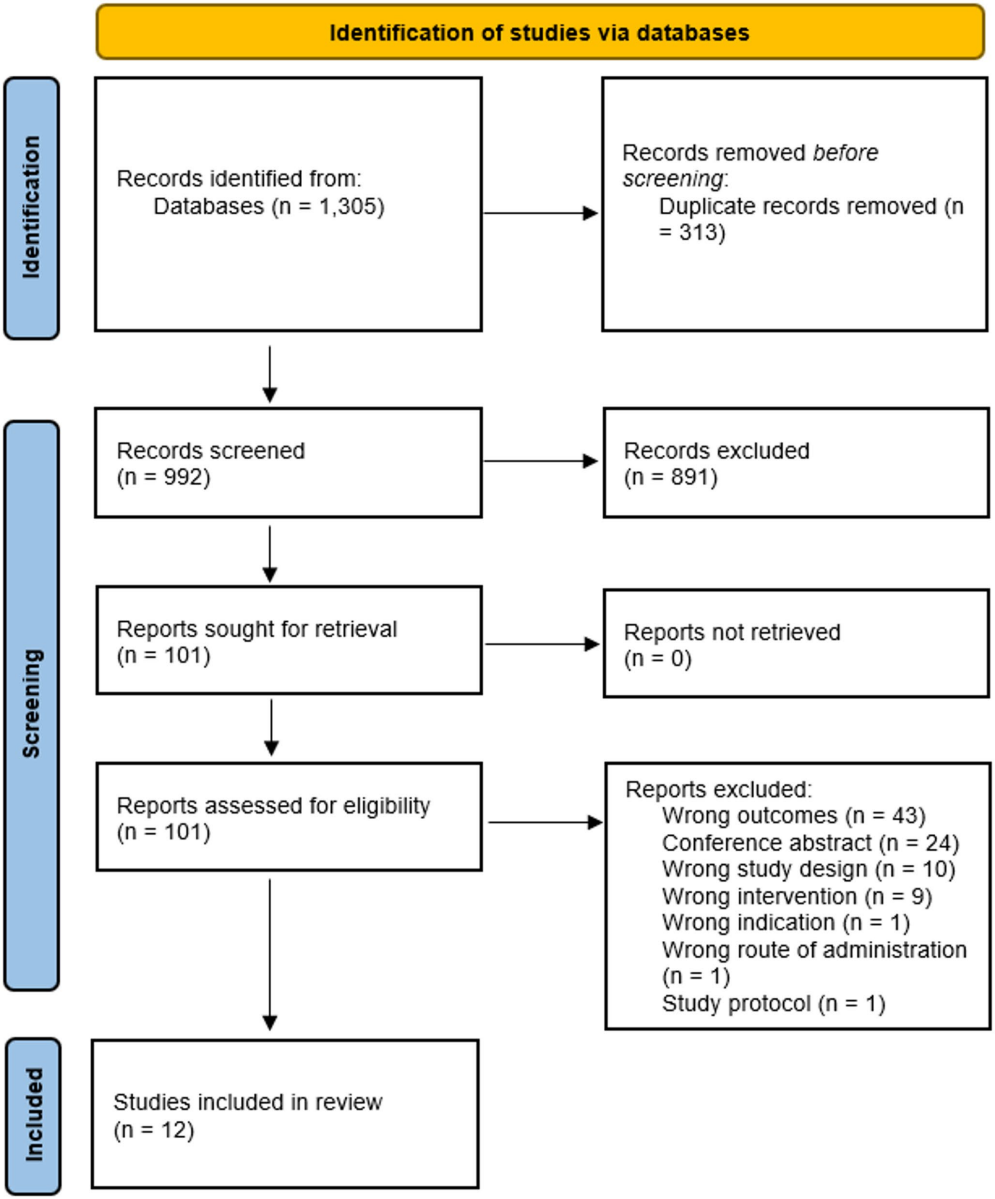
Flow diagram of study inclusion

The initial literature search identified 1,305 potential papers. After removal of duplicates, 992 articles were screened for inclusion based on their titles and abstracts, with 101 retrieved and subjected to full text screening. Finally, 12 articles (11 unique studies) met all inclusion criteria and were included [31–42]. The study selection procedure is shown in Fig. 1.

### Quality Assessment

Using the American Dietetic Association (ADA) quality assessment criteria (Figure S1), nine of the twelve included articles were rated as positive, high-quality trials [31, 33–39, 41] and three were graded as neutral [32, 40, 42].

### Characteristics of Included Studies

Twelve articles (11 unique studies) were included [31–42], with two from the same study [41, 42]. Six studies were randomised, cross-over trials [31, 34–36, 38, 41], four parallel [32, 37, 39, 40] and one a single arm pre-post intervention [33] (Table 2).

In terms of participants, only one study involved healthy adults exclusively (Table 2 and Table S3) [38]. The remaining studies included adults with pre-hypertension [32, 35, 36, 40, 41], stage one hypertension [32, 35, 40, 41], hypertensive black adults [31], or adults with risk factors for CVD [33, 34, 37, 39]. The proportion of female participants was similar or higher in all studies except for two [35, 42]. The number of study participants ranged from 14 [33] to 395 [40]. The studies were conducted in the USA (*n* = 6 [32, 34–36, 40, 41]), the United Kingdom (*n* = 3 [31, 38, 39]), China (*n* = 1 [33]), and Denmark (*n* = 1 [37]).

The duration of included articles varied from 3-weeks [38] to 32-weeks [41] including nine articles with a run-in period of between 6 days [35] and 3-weeks [40] (Table S3). The run-in period interventions varied between studies, such as instructions to maintain usual eating habits for 2-weeks [33, 36], having to eat two days of meals from each study diet [35], to consume the average Danish diet *ad libitum* except the last 3 days, when participants were provided specific Average Danish diet (ADD) foods in specific amounts to ensure that they were in energy balance [37], to follow a dietary guidance to reduce sodium intake for 2-weeks (2000 sodium mg/day) [31] or to consume 40 g of complex carbohydrate supplement [41, 42]. In the DASH sodium trial, all participants followed a high sodium diet during the run period (typical US consumption, 3450 sodium mg/day at 2100 kcal) [32, 40], while participants of the DASH trial during the run period followed a control diet (typical US consumption, 3000 sodium mg/day at 2100 kcal) [40].

**Table 2:** Summary of the included articles characteristics

	Study Design			Participants			Intervention		Metabolites			BP measurements method	
Ref, Year	Cross-over	Parallel	Pre-post intervention	Healthy adults	Adults with high BP, prehypertension or stage 1 HT	Adults with risk factors for CVD	Dietary Patterns	Specific food or nutrient	Bio-samples	Method used to assess metabolome	Targeted (T) Vs Un-targeted (U) Analysis	Office	Ambulant
Cheng, 2018 [32]		Y			Y		Y		Urine Blood	UPLC-MS and GC-MS (urine)	T	Unclear	Unclear
Loo, 2018 [35]	Y				Y		Y		Urine	^1^H-NMR	T&U	Y	
Chen, 2019 [31]	Y				Y			Y	Serum, Urine	UPLC-MS/MS (serum)	U	Y	Y
Reisdorph, 2020 [36]	Y				Y		Y		Urine	HPLC-MS	U		Y
Huang, 2021 [34]	Y					Y		Y	Plasma	UHPLC-ESI-MS/MS	T	Y	
Han, 2022 [33]			Y			Y	Y		Faeces, plasma	^1^H-NMR (faeces)	U	Y	
Qadir, 2022 [38]	Y			Y				Y	Plasma, urine and saliva	GC–MS for NO_3_^−^ (plasma, saliva and urine), and NO_2_^−^ (saliva) UHPLC-ESI-QqQ-MS/MS for plasma phenolic compounds	T		Y
Kim, 2023 [40]		Y			Y		Y		Urine Serum	UPLC-MS/MS	U	Y	
Sellem, 2023 [39]		Y				Y		Y	Plasma	CLP	T		Y
Trimigno, 2023 [37]		Y				Y	Y		Plasma	^1^H-NMR	U	Y	
Changwei, 2023* [41]	Y				Y			Y	Serum	UPLC-MS/MS	U	Y	
Sun, 2024* [42]	Y				Y			Y	Plasma	UPLC-MS/MS	U	Y	

*Belong to the same study. BP, blood pressure. CVD, cardiovascular disease. CLP, Complex Lipids Platform™. GC-MS, Gas chromatography- mass spectrometry. H-NMR, Proton nuclear magnetic resonance. HPLC-MS, high performance liquid chromatography- mass spectrometry. HT, hypertension. R, reference. UHPLC-ESI-MS/MS, ultra-high performance liquid chromatography–electrospray ionization tandem mass spectrometry. UHPLC-ESI-QqQ-MS/MS Ultra High-Performance Liquid Chromatography Electrospray Ionization Triple Quadrupole Mass Spectrometry. UPLC-MS: Ultra high-performance liquid chromatography- mass spectrometry

### Intervention

Six studies reported using a dietary pattern intervention [32, 33, 35–37, 40] (Table 2). Three studies tested the DASH diet [35, 36, 40]. The first was the DASH trial and the DASH-sodium trial, which randomised participants to 2 dietary patterns (DASH and control, typical USA diet) with different sodium levels (low 1150 mg/day, intermediate 2300 mg/day and high 3450 mg/day at 2100 kcal) [40]. The second study used a modified-DASH diet, Omnidiet, which involved modifying the standard DASH diet macronutrients to create three different dietary intervention: OmniProt (48% kcal from carbohydrate, 25% from protein, and 27% from fat), OmniCarb and OmniMFA (15% both from protein, 58% and 48% from carbohydrate and 27% and 37% from fat, respectively [35]. The third study used the DASH diet with different types of meat as the major protein source [36]. One study belongs to the DASH sodium trial, however the study was focused on the control arm (US typical diet) [32]. Of the three remaining studies, one implemented a diet based on the dietary habits from centenarians of Guangxi, China, characterised by high dietary fibre, low total energy, low fat, low protein, and low dietary cholesterol content [33]. The other compared the New Nordic Diet (NND) which was composed of higher intakes of organic foods, including whole grains, nuts, berries, fruit and vegetables, fish and seafood, and a lower meat content compared with the ADD (Table S3) [37].

Six interventions were not based on any dietary pattern (Table 2). These focused on specific foods and/or nutrient targets. One intervention focused on a low sodium diet (2000 mg sodium/day) versus a control [31], participants received advice on a low sodium diet in addition to prescription of 9 sodium pills (10 mmol sodium per tablet) or a placebo supplement per day to achieve a high sodium intake versus a low sodium intake, respectively [31]. Of the remaining articles, one was based on fat type [39] and four were based on a single food [34, 38, 41, 42], specifically beverages with added freeze-dried strawberry or placebo [34], lettuce enriched with differing nitrate and phenolic compound concentrations in a single lab-session [38], and a supplement of 40 g of soy protein, milk protein or carbohydrate in water or juice [41, 42].

Four intervention studies provided all the food to be consumed during the trial [32, 33, 35, 40]. In other studies participants received either all food except for dairy products [39] or coffee, tea and alcoholic beverages [37], a 7-day menu and some items [36], advice on how to implement the diet, including provision of a placebo or sodium supplement to consume [31], or a single food [34, 38, 41, 42]. The single food provided was freeze-dried strawberry beverage or a control beverage [34], while another study administered a single food along with low inorganic nitrate (NO3−) mineral water for consumption from the evening before until the end of the study visit, and a meal to consume at the evening prior to the intervention [38]. In the ProBP trial, participants were provided with soy protein, milk protein, and complex carbohydrate powders and were instructed to take the supplements twice per day in water or juice [41, 42].

Dietary intervention compliance was checked by completion of daily food diaries [32, 34, 35, 39], 24-hour urinary sodium excretion [31, 32], urinary excretion of urea nitrogen, potassium, and phosphorus [40], consumption of weekday lunches or dinners on site [32, 40], menu checklist [36], checking return of unconsumed packs [41, 42], or by communication with volunteers [33]. One paper, evaluated compliance in several ways, such as self-evaluated compliance and satisfaction [37].

### Outcome Measures

#### Changes in Blood Pressure Measurements in Response To Intervention

BP was most commonly measured using seated in-office measurement (*n* = 6 [33–35, 37, 40–42], using digital automatic BP device [31, 35], random-zero sphygmomanometer or sphygmomanometer [33, 40, 41], or not specify [34, 37]), followed by 24-hour ambulant measurement (*n* = 3 [36, 38, 39]), both (*n* = 1 [31]), or the method used is not clearly described (*n* = 1 [32]), (Table 2). Differences in the period, number of measurements and device used are summarised in Table S3. Two studies insufficiently reported details on the measures [33, 37].

Three articles identified a significant reduction in DBP post-intervention [31, 33, 35] and four studies reported significant reductions in SBP [31, 33, 35, 38]. One intervention did not show any difference in BP [34] and seven articles did not report the difference in BP or whether any observed differences were statistically significant [36, 37, 39–42] or were unclear [32] (Table S4).

#### Sample Collection and Metabolome Qualification

Metabolites were identified in urine [32, 35, 36], faeces [33], saliva [38], plasma [34, 37, 39, 42], serum [31, 41] or in plasma/serum and urine [38, 40], respectively. The metabolome was quantified using Proton Nuclear Magnetic Resonance (^1^H-NMR) [33, 35, 37], High [36], Ultra [31, 32, 40–42], or Ultra-High [34, 38] Performance Liquid Chromatography-Mass Spectrometry (HPLC-MS, UPLC-MS, UHPLC-MS, respectively), Complex Lipid Platform (CLP) [39] or Gas Chromatography-Mass Spectrometry (GC-MS) [32, 38] (Table 2).

#### Relationship between Metabolites and BP

More than 100 unique metabolites were linked to BP. Of these, 40 were associated with SBP, 29 with DBP, 31 with both SBP and DBP, and 2 did not specify whether they were associated with SBP or DBP (Table 3 and Table S4). Several metabolite super pathways such as amino acids (*n* = 26), carbohydrates (*n* = 3), cofactors and vitamins (*n* = 10), energy (*n* = 1), lipids (*n* = 24), nitric acid (*n* = 1), nucleotides (*n* = 3), peptides (*n* = 5) and xenobiotics (*n* = 10) were linked to BP outcomes, along with more than 40 different sub-pathways (Table 3). However, the pathway for some metabolites were unknown (*n* = 19) or only identified by their molecular weights as the exact metabolite was not identified (*n* = 9). The metabolite profiles demonstrated positive (*n* = 38), negative (*n* = 48) and both (*n* = 2) associations with BP (Table 3). For 15 metabolites, an association with BP was reported without specifying the direction of the relationship. Only two metabolites identified were found to have a significant relationship with BP measures across multiple studies (Table 3), these were N-acetylneuraminate [31, 36], showing negative or no direct relationship with BP, and proline-betaine, also known as stachydrine, which had a negative association with BP in both papers [35, 40].

**Table 3:** Summary of reported statistically significant metabolite associations with blood pressure outcomes, categorised by super pathways and sub-pathways from the included articles

Metabolites	REF	DIET	Super Pathway^§^		Sub Pathway^§^	SBP/DBP	Association	Metabolome method	Biofluid
Β-hydroxyisovalerate	[31]	Sodium Reduction	Amino Acid	Leucine, Isoleucine and Valine Metabolism		SBP	-	UPLC-MS	Serum
N2-acetyllysine	[31]	Sodium Reduction	Amino Acid	Lysine metabolism		SBP/DBP	-	UPLC-MS	Serum
Methionine sulfone	[31]	Sodium Reduction	Amino Acid	Methionine, Cysteine, SAM and Taurine Metabolism		DBP	-	UPLC-MS	Serum
Vanillactate	[31]	Sodium Reduction	Amino Acid	Tyrosine Metabolism		DBP	-	UPLC-MS	Serum
Lysine	[32]	Either low or high sodium	Amino Acid	Lysine metabolism		SBP	+	UPLC-MS/GC-MS	Urine
Cystine	[32]	Either low or high sodium	Amino Acid	Methionine, Cysteine, SAM and Taurine Metabolism		SBP/DBP	+	UPLC-MS/GC-MS	Urine
Citrulline	[32]	Either low or high sodium	Amino Acid	Urea cycle; Arginine and Proline Metabolism		SBP	+	UPLC-MS/GC-MS	Urine
Alanine	[33]	Derived from centenarians Guangxi	Amino Acid	Alanine and Aspartate Metabolism		SBP	+	H-NMR	Faeces
Aspartate	[33]	Derived from centenarians Guangxi	Amino Acid	Alanine and Aspartate Metabolism		SBP/DBP	+	H-NMR	Faeces
L-glutamic acid	[36]	DASH	Amino Acid	Glutamate Metabolism		SBP/DBP		HPLC-MS	Urine
3-indolebutyric acid	[36]	DASH	Amino Acid	Tryptophan Metabolism		SBP/DBP	-	HPLC-MS	Urine
3-(4-hydroxyphenyl) lactate	[40]	DASH- *control high-sodium*	Amino Acid	Tyrosine Metabolism		DBP	+	UPLC-MS/MS	Urine
3,4-dihydroxyphenylacetate sulfate	[40]	DASH *-High*,* low and control Sodium*	Amino Acid	Tyrosine Metabolism		SBP/DBP	+/-	UPLC-MS/MS	Urine
Dopamine 3-o-sulfate	[40]	DASH *-High Sodium*	Amino Acid	Tyrosine Metabolism		SBP/DBP	-	UPLC-MS/MS	Urine
Dopamine 4-sulfate	[40]	DASH *-High Sodium*	Amino Acid	Tyrosine Metabolism		DBP	-	UPLC-MS/MS	Urine
N, n-dimethyl-5-aminovalerate	[40]	DASH *-High Sodium*	Amino Acid	Lysine Metabolism		SBP	-	UPLC-MS/MS	Urine
N, n-dimethylalanine	[40]	DASH- *control high-sodium*	Amino Acid	Alanine and Aspartate Metabolism		SBP	-	UPLC-MS/MS	Urine
N-carbamoylvaline	[40]	DASH *-High Sodium and control*	Amino Acid	Leucine, Isoleucine and Valine Metabolism		SBP	-	UPLC-MS/MS	Urine
N-methylglutamate	[40]	DASH*-Low sodium*	Amino Acid	Glutamate Metabolism		SBP	-	UPLC-MS/MS	Urine
N-methylhydroxyproline	[40]	DASH*-Low and high sodium*	Amino Acid	Urea cycle; Arginine and Proline Metabolism		SBP	-	UPLC-MS/MS	Urine
N-methylproline	[40]	DASH *-High Sodium*	Amino Acid	Urea cycle; Arginine and Proline Metabolism		DBP	-	UPLC-MS/MS	Urine
Tryptophan betaine	[40]	DASH	Amino Acid	Tryptophan Metabolism		DBP	-	UPLC-MS/MS	Serum
Isobutyrylglycine	[41]	ProBP-Soy	Amino Acid	Leucine, Isoleucine and Valine Metabolism		SBP/DBP	-	UPLC-MS/MS	Serum
Isovalerylglycine	[41]	ProBP-Soy	Amino Acid	Leucine, Isoleucine and Valine Metabolism		SBP/DBP	-	UPLC-MS/MS	Serum
Alpha-ketobutyrate	[42]	ProBP-milk	Amino Acid	Methionine, Cysteine, SAM and Taurine Metabolism		SBP	+	UPLC-MS/MS	Plasma
N-acetyltyrosine	[42]	ProBP-Carb	Amino Acid	Tyrosine Metabolism		DBP	+	UPLC-MS/MS	Plasma
N-acetylneuraminate	[31]	Sodium Reduction	Carbohydrate	Aminosugar Metabolism		SBP/DBP	-	UPLC-MS	Serum
	[36]	DASH	Carbohydrate	Aminosugar Metabolism		DBP		HPLC-MS	Urine
Sucrose	[31]	Sodium Reduction	Carbohydrate	Disaccharides and Oligosaccharides		SBP/DBP	+	UPLC-MS	Serum
Maltose	[42]	ProBP-soy	Carbohydrate	Glycogen Metabolism		DBP	+	UPLC-MS/MS	Plasma
N-methyl-2-pyridone-5-carboxamide	[35]	OmniCarb	Cofactors and Vitamins	Nicotinate and Nicotinamide Metabolism		SBP/DBP	-	H-NMR	Urine
1-(beta-d-ribofuranosyl) − 1,4-dihydronicotinamide	[36]	DASH	Cofactors and Vitamins	Nicotinate and Nicotinamide Metabolism		SBP	+	HPLC-MS	Urine
Pantothenate (vitamin b5)	[40]	DASH*-Low and high sodium*	Cofactors and Vitamins	Pantothenate and CoA Metabolism		SBP/DBP	-	UPLC-MS/MS	Urine
Carotene diol (3)	[40]	DASH	Cofactors and Vitamins	Vitamin A Metabolism		SBP	+	UPLC-MS/MS	Serum
Pyridoxate	[40]	DASH*-High Sodium*	Cofactors and Vitamins	Vitamin B6 Metabolism		SBP	-	UPLC-MS/MS	Urine
Alpha-cehc glucuronide	[40]	DASH *-High and Low Sodium*	Cofactors and Vitamins	Tocopherol Metabolism		SBP/DBP	-	UPLC-MS/MS	Urine
Alpha-cehc taurine	[40]	DASH *-High and Low Sodium*	Cofactors and Vitamins	Tocopherol Metabolism		SBP	-	UPLC-MS/MS	Urine
Thiamine (vitamin b1)	[40]	DASH*-High Sodium*	Cofactors and Vitamins	Thiamine Metabolism		DBP	-	UPLC-MS/MS	Urine
Riboflavin (vitamin b2)	[40]	DASH -*High Sodium*	Cofactors and Vitamins	Riboflavin Metabolism		DBP	-	UPLC-MS/MS	Urine
Alpha- cehc sulfate	[42]	ProBP-soy	Cofactors and Vitamins	Tocopherol Metabolism		SBP	+	UPLC-MS/MS	Plasma
Succinic acid	[37]	NND	Energy	TCA Cycle		DBP	-	H-NMR	Plasma
Campesterol	[31]	Sodium Reduction	Lipid	Sterol		SBP/DBP	+	UPLC-MS	Serum
Carnitine	[35]	OmniProt	Lipid	Carnitine Metabolism		SBP	+	H-NMR	Urine
3-hydroxybutyric acid	[37]	NND	Lipid	Ketone bodies		DBP	-	H-NMR	Plasma
Acetoacetic acid	[37]	NND	Lipid	Ketone bodies* from the paper		DBP	-	H-NMR	Plasma
Acetone	[37]	NND	Lipid	Ketone bodies* from the paper		DBP	-	H-NMR	Plasma
1-palmitoyl-2-oleoyl-gpc (16:0/18:1)	[40]	DASH	Lipid	Phosphatidylcholine		SBP/DBP	+	UPLC-MS/MS	Serum
1-stearoyl-2-oleoyl-gpc (18:0/18:1)	[40]	DASH	Lipid	Phosphatidylcholine		SBP	+	UPLC-MS/MS	Serum
Glycosyl-n-palmitoyl-sphingosine (d18:1/16:0)	[40]	DASH	Lipid	Hexosylceramides		SBP/DBP	+	UPLC-MS/MS	Serum
Glycosyl-n-stearoyl-sphingosine	[40]	DASH and control	Lipid	Hexosylceramides		SBP	+/-	UPLC-MS/MS	Serum
Picolinoylglycine	[40]	DASH *-High Sodium*	Lipid	Fatty Acid Metabolism (Acyl Glycine)		SBP	-	UPLC-MS/MS	Urine
Erucate	[41]	ProBP**	Lipid	Long Chain Monounsaturated Fatty Acid		SBP	+	UPLC-MS/MS	Plasma
Dihomo-linolenoyl-choline	[41]	ProBP soy and milk	Lipid	Fatty Acid Metabolism (Acyl Choline)		BP	-	UPLC-MS/MS	Plasma
Oleoylcholine	[41]	ProBP soy and milk	Lipid	Fatty Acid Metabolism (Acyl Choline)		BP	-	UPLC-MS/MS	Plasma
1-linoleoyl-gpe (18:2)	[42]	ProCarb	Lipid	Lysophospholipid		DBP	+	UPLC-MS/MS	Plasma
1-oleoyl-gpe (18:1)	[42]	ProCarb	Lipid	Lysophospholipid		DBP	+	UPLC-MS/MS	Plasma
1-stearoyl-2-linoleoyl-gpc (18:0/18:2)	[42]	ProCarb	Lipid	Phosphatidylcholine		DBP	+	UPLC-MS/MS	Plasma
1-palmitoyl-2-oleoyl-gpe (16:0/18:1)	[42]	ProCarb	Lipid	Phosphatidylethanolamine		DBP	+	UPLC-MS/MS	Plasma
1-stearoyl-2-linoleoyl-gpe (18:0/18:2)	[42]	ProCarb	Lipid	Phosphatidylethanolamine		DBP	+	UPLC-MS/MS	Plasma
1-stearoyl-2-oleoyl-gpe (18:0/18:1)	[42]	ProCarb	Lipid	Phosphatidylethanolamine		DBP	+	UPLC-MS/MS	Plasma
Glycerol	[42]	ProMilk	Lipid	Glycerolipid Metabolism		SBP	+	UPLC-MS/MS	Plasma
Hexadecadienoate (16:2n6)	[42]	ProMilk	Lipid	Long Chain Polyunsaturated Fatty Acid (n3 and n6)		SBP	+	UPLC-MS/MS	Plasma
Palmitoyl-arachidonoyl-glycerol (16:0/20:4) [2]	[42]	ProMilk	Lipid	Diacylglycerol		SBP	+	UPLC-MS/MS	Plasma
N-stearoyl-sphinganine (d18:0/18:0)	[42]	ProSoy	Lipid	Dihydroceramides		SBP	+	UPLC-MS/MS	Plasma
5-hepe	[42]	ProSoy	Lipid	Unknow		SBP	+	UPLC-MS/MS	Plasma
No_3_^−^	[38]	HNLP	Nitric Acid	Nitrogen Metabolism		SBP	-	UPLC-MS/MS	Saliva
β-aminoisobutyric acid	[32]	Either low or high sodium	Nucleotide	Pyrimidine Metabolism, Thymine containing		SBP	-	UPLC-MS/GC-MS	Urine
Allantoin	[40]	DASH *-High Sodium*	Nucleotide	Purine Metabolism, (Hypo)Xanthine/Inosine containing		SBP	-	UPLC-MS/MS	Urine
N6-carbamoylthreonyladenosine	[42]	ProSoy	Nucleotide	Purine Metabolism, Adenine containing		SBP	+	UPLC-MS/MS	Plasma
Phenylacetylglutamine	[35]	OmniMFA*	Peptide	Acetylated Peptides		DBP	-	H-NMR	Urine
N-(phenylacetyl)glutamic acid	[36]	DASH	Peptide	Acetylated Peptides		SBP		HPLC-MS	Urine
3-hydroxyphenyl acetoylglutamine	[40]	DASH *-High and Low Sodium*	Peptide	Acetylated Peptides article		SBP	-	UPLC-MS/MS	Urine
Gamma-glutamyl-alpha-lysine	[40]	DASH*- control high-sodium*	Peptide	Gamma-glutamyl Amino Acid art		SBP	-	UPLC-MS/MS	Urine
Phenylacetylglycine	[40]	DASH *-High Sodium*	Peptide	Acetylated Peptides		SBP/DBP	-	UPLC-MS/MS	Urine
Homocysteine	[32]	Either low or high sodium	Unknown			SBP	+	UPLC-MS/GC-MS	Urine
2-acetyl-3-methylpyrazine	[36]	DASH	Unknown			SBP/DBP		HPLC-MS	Urine
2-(3-methylthiopropyl)malate	[36]	DASH	Unknown			SBP/DBP		HPLC-MS	Urine
3-(3-methylbutylidene)-1(3 h)-isobenzofuranone	[36]	DASH	Unknown			DBP		HPLC-MS	Urine
Bicine	[36]	DASH	Unknown			DBP		HPLC-MS	Urine
Potassium gluconate	[36]	DASH	Unknown			DBP		HPLC-MS	Urine
Kynuramine	[36]	DASH	Unknown			SBP	-	HPLC-MS	Urine
Physoperuvine	[36]	DASH	Unknown			SBP/DBP	-	HPLC-MS	Urine
Val-glu	[36]	DASH	Unknown			SBP/DBP		HPLC-MS	Urine
Lpc (22:5)	[39]	DIVAS	Unknown			SBP	+	CLP	Plasma
4-cresyl sulfate	[35]	OmniMFA*	Xenobiotics	Benzoate Metabolism		SBP/DBP	-	H-NMR	Urine
Hippurate	[35]	OmniCarb	Xenobiotics	Benzoate Metabolism		SBP/DBP	+	H-NMR	Urine
Proline-betaine	[35]	OmniCarb diet and MFA	Xenobiotics	Food Component/Plant		SBP/DBP	-	H-NMR	Urine
	[40]	DASH-*low and high sodium*	Xenobiotics	Food Component/Plant		SBP/DBP	-	UPLC-MS/MS	Urine
2,8-quinolinediol sulfate	[40]	DASH *-High Sodium*	Xenobiotics	Food Component/Plant		DBP	-	UPLC-MS/MS	Urine
3-hydroxyindolin-2-one	[40]	DASH*- control high-sodium*	Xenobiotics	Food Component/Plant		DBP	+	UPLC-MS/MS	Urine
3-hydroxystachydrine	[40]	DASH*-low sodium*	Xenobiotics	Food Component/Plant		SBP	-	UPLC-MS/MS	Urine
4-hydroxycinnamate	[40]	DASH*- control high-sodium*	Xenobiotics	Food Component/Plant		SBP/DBP	+	UPLC-MS/MS	Urine
4-hydroxymandelate	[40]	DASH *-High Sodium*	Xenobiotics	Benzoate Metabolism		DBP	-	UPLC-MS/MS	Urine
N-acetylalliin	[40]	DASH*- control high-sodium*	Xenobiotics	Food Component/Plant		SBP/DBP	+	UPLC-MS/MS	Urine
3-indoleglyoxylic acid	[42]	ProBP Pretrial baseline	Xenobiotics	Food Component/Plant		DBP	+	UPLC-MS/MS	Plasma
X-23,276	[42]	ProCarb	Unknown			SBP	-	UPLC-MS/MS	Plasma
Undefined with mass 73.0264	[36]	DASH	Unknown			SBP		HPLC-MS	Urine
Undefined with mass 121.917	[36]	DASH	Unknown			SBP/DBP		HPLC-MS	Urine
Undefined with mass 124.039	[36]	DASH	Unknown			SBP/DBP		HPLC-MS	Urine
Undefined with mass 238.1336	[36]	DASH	Unknown			SBP/DBP		HPLC-MS	Urine
Undefined with mass 268.1409	[36]	DASH	Unknown			SBP/DBP		HPLC-MS	Urine
Undefined with mass 291.0951	[36]	DASH	Unknown			DBP		HPLC-MS	Urine
Undefined with mass 265.0971	[36]	DASH	Unknown			SBP	-	HPLC-MS	Urine
Undefined with mass 157.0373	[36]	DASH	Unknown			SBP	-	HPLC-MS	Urine

The empty cells in the association column are due to the lack of information on the direction of the association type in the original article. * vs. OmniProt and not vs. baseline than others. ^§^ Super and sub-pathway data was obtained from the article, Metabolon Inc and Human Metabolome Database [43]. CLP, Complex Lipids Platform. CoA, coenzyme A. DBP, diastolic blood pressure. H-NMR, Proton Nuclear magnetic Resonance. HPLC-MS, High-Performance Liquid Chromatography-Mass Spectrometry. LPC, lysophosphatidylcholine SAM, S-adenosylmethionine. SBP, systolic blood pressure. TCA cycle, tricarboxylic. UPLC-MS/MS, Ultra-Performance Liquid Chromatography-Tandem Mass Spectrometry. GC-MS, Gas Chromatography-Mass Spectrometry acid cycle

#### Association between Change in BP and Metabolites Included in Outcome Measures

Nine studies reported results specifically examining the association between change in BP (in response to diet) and metabolites [31, 34–41]. Eight studies reported significant associations [31, 35–41]. One paper examined the associations with BP and metabolites and also associations with change in BP [36] and in three [32, 33, 42] it was unclear whether the association reported was with the change in BP or with BP itself (Table 3 and Table S4). One paper reported no significant correlation between metabolites and change in BP [34].

Regarding dietary interventions, four of the eight studies that reported significant associations between change in BP and metabolites response implemented a whole dietary pattern intervention [35–37, 40]. Among these, three were variations of the DASH diet [35, 36, 40] and one followed the New Nordic Diet (NND) [37]. Following the OmniDiet, a variation of the DASH diet, change in BP was significantly associated with six urinary metabolites. These included markers of dietary intake (proline-betaine (inverse) and carnitine (direct)), gut microbial co-metabolites (hippurate (direct), 4-cresyl sulfate (inverse), phenylacetylglutamine (inverse), and a metabolite related to tryptophan metabolism (N-methyl-2-pyridone-5-carboxamide (inverse) [35]. Another study based on the DASH dietary pattern found that 16 metabolites were associated with BP and six were associated with change in BP over time [36]. Meanwhile, Kim et al., identified 42 metabolites, among which several significantly differentiated between the DASH and control diet interventions, including serum tryptophan betaine (DASH trial), urine N-methylglutamate and urine proline derivatives such as stachydrine, 3-hydroxystachydrine, N-methylproline, and N-methylhydroxyproline (DASH-Sodium trial). These metabolites were associated with reductions in SBP or DBP in the DASH diets intervention, but not in the control diet. Additionally, they performed a secondary analysis using elastic net linear regression to identify metabolites predicting BP. In the DASH trial, six serum metabolites were associated with changes in SBP: tryptophan betaine and β-cryptoxanthin (not previously identified) were associated with SBP reductions, while the remaining metabolites (1-oleoyl-GPC [18:1] (not previously identified), 1-stearoyl-2-oleoyl-GPC [18:0/18:1], 1-[1-enyl-stearoyl]-2-linoleoyl-GPE [P-18:0/18:2] (not previously identified), and theobromine) were associated with SBP elevations. In the DASH-Sodium trial, five metabolites were associated with BP changes, with three (3,5-dihydroxybenzoic acid (not previously identified), N-methylglutamate, and stachydrine) associated with BP reductions, while theobromine and 3-methylxanthine (not previously identified) were associated with BP increases [40]. In addition, although tryptophan betaine, 2-hydroxysebacate, abscisate, carnosine, 5-hydroxymethyl-2-furoic did not differ within each diet, concentrations of these metabolites were significantly different between intervention groups in the DASH-Sodium trial (*p* < 0.050). These new metabolites were not included in the Table 3 due to differences in data presentation in the original paper and the current focus on the primary findings. The remaining study, which involved a whole dietary pattern intervention (New Nordic Diet), identified four metabolites that were inversely associated with the increase in DBP in the NND group [37] (Table 3 and Table S4).

On the other hand, four studies were not based on dietary pattern interventions, but used specific nutrient or food interventions. In a sodium reduction intervention Chen et al., [31] identified changes in 34 metabolites (*p* < 0.05), with the top 10 reported having stronger associations (*p* < 0.01) than the others. Six of these metabolites were upregulated by sodium reduction, including β-hydroxyisovalerate (HMB) and methionine sulfone, which remained significant after FDR correction (FDR = 0.006, 0.099, respectively). Increased HMB was associated with reduced office and ambulatory daytime SBP, while increased methionine sulfone was linked to reduced 24-hour and ambulatory nighttime DBP [31]. Two studies showed, association between one metabolite and change in SBP [38] and night SBP [39]. The results of Changwei et al., [41] demonstrated that changes in erucate were positively associated with SBP changes and higher odds of hypertension. Additionally, high baseline levels of acylcholines dihomolinolenoyl-choline and oleoylcholine were predictive of greater BP reduction following soy protein consumption. In contrast, increased cheese intake during the trial, as reflected by isobutyrylglycine and isovalerylglycine, attenuated the blood pressure-lowering effect of soy protein (Table 3 and Table S4).

## Discussion

The current systematic review summarises findings from 12 papers (11 unique studies) on the relationship between diet, metabolites and BP change associated with nutritional metabolomics. Ten unique studies identified significant associations between 102 unique diet-related metabolites and BP, of which 40 were associated with SBP, 29 with DBP, 31 with both SBP and DBP, and 2 did not specify whether they were associated with SBP or DBP. However, only two metabolites, proline betaine (stachydrine) and N-acetylneuraminate, were found to have a significant relationship with BP measures, each identified in two independent studies.

Dietary interventions used in the included studies were heterogenous, contributing to the heterogeneity in results. Although identifying biomarkers of dietary patterns could contribute further to our understanding of the relationship between diet and disease [22, 44–46], dietary patterns were only reported in six publications. Three of the included dietary patterns were based on the DASH diet, however definitions varied between studies including variations in sodium content, source of protein or percentage of total energy contributed by each macronutrient of each diet. The use the DASH dietary pattern is not surprising since meta-analyses indicate it significantly lowers BP in both hypertensive and normotensive adults [6, 47, 48]. The heterogeneity across the different DASH diets included in the current review, may have limited the strength and consistency of findings.

Of the four papers administering a single food intervention [34, 38, 41, 42], two papers (same study) used soy and milk protein supplements [41, 42] and two studies used high polyphenol foods, strawberries [34] and polyphenol-fortified lettuce [38]. One of these studies found a relationship between nitrate-enriched lettuce and reduced SBP [38]. Nitrate intake and the salivary microbiome have been shown to have vasodilating effects and may have a role in reducing BP [49–51]. Therefore, it is not surprising that salivary nitrate levels after the consumption of nitrate enriched lettuce resulted in a reduction in BP. On the other hand, it is important to highlight that in the cross-over study, where a beverage made from freeze-dried strawberries was administered to one arm and a placebo beverage to the other, no statistically significant relationship between metabolites and BP was found [34]. This is unexpected as consuming high polyphenol foods have been shown to result in a reduction in BP [52–54] and polyphenols have been expressed in biofluids as a marker of polyphenol intake [55]. A limitation of single food studies is that they do not account for the synergistic effects between food and nutrient intakes within the whole diet [47]. Additionally, there is the potential for a dose-response effect which needs to be considered. A recent review concluded that evidence from human studies suggests that some polyphenol-rich foods exert positive effects on blood pressure levels. However, the authors highlight that, given the limited clinical effects reported, the real-world implications of consumption will depend on their inclusion within a healthy diet pattern, rather than the consumption of an individual food [56]. Additionally, two studies using a single food [34, 38] used targeted metabolomic approaches to evaluate associations with BP and therefore may not represent the broad range of metabolites that could potentially influence BP regulation. This could explain the lack of relationship demonstrated between metabolites and BP arising from these foods in these studies.

The absence of comparable metabolites associated with BP across the various studies may be attributed to various factors such as variation in the dietary interventions used, biofluids sampled or methods used to quantify the diet-related metabolome [22]. Several different biofluids were used in the studies: plasma (*n* = 5), urine (*n* = 5), serum (*n* = 3), faeces (*n* = 1) and saliva (*n* = 1). Although urinary metabolite concentrations can be variable due to factors such as ionic strength, pH, osmolarity, and dilution [57], higher concentrations of dietary compounds and wider dynamic ranges have been found in urine compared to blood samples [58]. Therefore, the collection of both blood and urine samples in the same study can provide complementary information about food digestion and metabolism [59]. However, the critical question of which biospecimen generates a range of metabolite biomarkers that can best characterise habitual diet remains unanswered.

Regarding metabolite analysis, different methods were used across the studies: UPLC-MS, GC-MS, HPLC-MS, UHPLC-MS and H-NMR. The discrepancies observed among the studies included may be due to the intrinsic differences in the use of these methods [60, 61]. In the current review, LC-MS is the predominant method. This finding is similar to that found in a review of methods assessing the dietary metabolome, which found LC-MS methods to be the primary method used to analyse the metabolome [22]. Due to its high sensitivity and versatility, allowing the detection of a wide range of organic and inorganic compounds, the UPLC system is currently the main technology used in metabolomics research [61]. However, LC-MS has certain limitations, such as problems related to sample recovery and cost [60–63]. Moreover, metabolomics can be targeted or untargeted [64, 65]. Untargeted methods were used in more than a half of the included articles. This method is focused on requiting data for as many metabolites as possible, for both known and unknown metabolites [64, 65]. This data can be used for relative quantification and to provide hypotheses that can be further studied with targeted approaches and is used widely in nutritional metabolomic studies [64, 65]. Since the field continues to emerge and techniques are evolving very rapidly, the variation in methodologies between studies is to be expected.

Only two metabolites demonstrated reproducible associations with blood pressure, each identified in two independent studies. The first, proline-betaine, also known as stachydrine, is a xenobiotic compound found in high concentrations in citrus fruits [66]. It has emerged as a promising biomarker of citrus intake [66, 67] and an indicator of specific healthy dietary patterns such as the Mediterranean [68] and DASH diets [69]. Of note, in two of the included studies testing the DASH diet, a statistically significant negative association was identified between this metabolite and SBP and DBP [35, 40]. These results are in line with a recent study that found negative associations between proline-betaine and plasma lipid profiles, BMI, fasting insulin, high-sensitivity C-reactive protein, smoking, and alcohol intake, which suggests a potential role of diet in prevention and /or treatment of CVD [70]. Stachydrine increases the production of nitric oxide [71], an important vasodilator that contributes to the relaxation of blood vessels [72, 73], which may lead to a decrease in BP.

The second metabolite, N-acetylneuraminate demonstrated conflicting results, with one study reporting a negative association with BP [31], while the other study did not mention the direction of association [36]. The N-acetylneuraminate is the conjugate base of N-acetylneuraminic acid [74], however these terms are commonly treated as synonymous [43]. This compound is one of the most abundant sialic acids in humans and is derived from the amino sugar neuraminic acid [43, 74]. Sialic acids are important in the regulation of host immunity [75]. Previous literature identified this metabolite as a significant detrimental risk factor for CVD when its concentration is elevated, although its relationship with BP is unclear [76, 77]. While some animal studies suggest potential vascular effects, including vasodilation, the evidence is not yet conclusive [78, 79]. More research is needed on this metabolite, taking into account genetics and ethnicity, given its potential relevance in BP regulation [80].

While the findings regarding specific metabolites have not yielded conclusive results across various studies, a different pattern emerges when considering metabolic pathways. Notably, seven of the included articles reported the presence of specific metabolites within the metabolic pathway of amino acids. Moreover, a significant number of the approximately 30 metabolites associated with blood pressure reduction belong to the amino acid class. These compounds are bioactive peptides derived from dietary proteins and may be relevant in the pathways involved in BP regulation by potentially inhibiting angiotensin-converting enzymes [81].

In recent years, research examining the relationship between amino acids and hypertension has increased, although conclusive evidence has not yet been established due to the discrepancies found in the literature [82]. For instance, one included study found an unspecified association between L-glutamic acid and diet [36], however another reported a favourable relationship between dietary glutamic acid and BP, indicating that an elevated dietary intake was associated with lower values of SBP and DBP [83]. Interestingly, this observation contradicts the results of another study which found no significant association between dietary glutamic acid, BP levels, or the incidence of hypertension [84]. Further studies are currently needed to understand the relationship between amino acids and BP regulation.

The potential contribution of the gut microbiota to production of specific metabolites included in this review, such as hippurate, 4-cresyl sulfate and phenylacetylglutamine should be noted [46, 85]. The intestinal microbiota can adapt to dietary alterations and cause changes in host metabolism [86]. In previous literature it was reported that hippurate was strongly associated with increased gut microbial diversity and that consumption of whole grains, fruit and polyphenol-rich foods was associated with reduced odds of metabolic syndrome [87]. The intestinal microbiota also plays a crucial role in the metabolism of dietary polyphenols and in influencing BP regulation [88, 89]. Recent research found positive correlations between higher intake of berry flavonoids and lower SBP [89], with approximately 12% of the cardioprotective benefits attributed to changes in the gut microbiota [89]. These findings highlight the importance of simultaneously quantifying the microbiome as a possible intermediary in the relationship between diet, BP, metabolic status and health.

### Strengths and Limitations

Notably, six of the eleven unique studies were cross-over trials, with each participant serving as their own control, reducing between-person variations. Seven studies provided most of the food consumed during the intervention, minimising variations caused by differences in food intake. In addition, compliance with the diet was measured in eleven studies, with three studies using an objective 24-h urinary excretion of known metabolites as a marker of compliance. The use of validated tools and objective measures of dietary compliance can further enhance our understanding of diet-disease relationships. However, this review also has some limitations. Firstly, the number of included studies was relatively small (*n* = 11) and the substantial heterogeneity in dietary interventions, ranging from overall dietary patterns to specific foods. These factors, along with differences in study duration (between, approximately 3 to 32 weeks), BP measurement, biological samples, analysis techniques (targeted versus untargeted) and statistical analysis used to measure metabolites varied between studies, limiting comparability, and thus potentially leading to the differences observed being due to varying methodologies implemented. Additionally, although similar super pathways were measured in all articles (e.g. Amino Acid), the same metabolites were not always studied or reported. With over 100 metabolites reported and limited replication across studies, concerns arise regarding both specificity and biological plausibility. All these factors may affect the findings and restrict the generalizability of the conclusions at this time.

### Recommendations

A significant proportion of studies that assessed dietary metabolites and impact on BP did not explore the direct relationship between these variables, resulting in the exclusion of 19 articles during the screening process. Future studies could consider contributing evidence to better understand the relationship between metabolites and BP by incorporating both targeted and untargeted metabolomic methods to help validate key metabolites and aid in the identification of new metabolites. Although, few similar metabolites were identified in the current review, it can serve as a starting point for targeted metabolomics, by examining nutritional pathways that are known to play a role in BP regulation and targeting known metabolites within those pathways for future investigation.

## Conclusion

The current review underlines the importance of diet-related metabolites in BP management, highlighting the potential of nutritional metabolomics as a promising approach to BP control and CVD risk reduction through precision nutrition. Nevertheless, further research is essential to gain a deeper understanding of the underlying mechanisms, including the specific metabolites and pathways involved, as well as the particular aspects of diet that play a role.

## Key References

Brennan L, de Roos B. Role of metabolomics in the delivery of precision nutrition. Redox Biology. 2023;65:102808. doi: 10.1016/j.redox.2023.102808.

This review focuses on key concepts and studies using metabolomics/biomarker measurements and explores areas where metabolomics has shown a key role including biomarkers of food intakes, metabolic phenotyping and response to interventions.

Clarke ED, Ferguson JJ, Stanford J, Collins CE. Dietary assessment and metabolomic methodologies in human feeding studies: a scoping review. Advances in Nutrition. 2023. doi: 10.1016/j.advnut.2023.08.010.

The review emphasises the importance of establishing reporting guidelines for diet-related metabolome studies to enhance comparability and reproducibility. It proposes a checklist for researchers to consider when reporting on the diet and methodologies used.

Jama HA, Snelson M, Schutte AE, Muir J, Marques FZ. Recommendations for the use of dietary fiber to improve blood pressure control. Hypertension. 2024;81(7):1450-9. doi: 10.1161/HYPERTENSIONAHA.123.22575.

This paper reviews evidence showing that increased fiber intake supports a healthy gut microbiota and the production of short-chain fatty acids, microbiota-derived metabolites known to lower BP, offering practical guidance for improving cardiovascular outcomes through diet.

## Electronic Supplementary Material

Below is the link to the electronic supplementary material.

Supplementary Material 1

## Data Availability

No datasets were generated or analysed during the current study.
