# Supplementary material for: Metabolite Profiles in Response to Dietary Interventions for Management of Blood Pressure: A Systematic Review
Source: Curr Nutr Rep. 2025 Jun 20;14(1):82. doi: 10.1007/s13668-025-00676-7 (PMC12181105; doi:10.1007/s13668-025-00676-7)
Supplement: Supplementary file 1 — Supplementary Material 1 [file 13668_2025_676_MOESM1_ESM.docx]

**Supplementary data**

**Metabolite profiles in response to dietary interventions for management of blood pressure: a systematic review**

**Authors name:**

María Gómez-Martín,^1,2^ PhD.

Floor Rikken,^3^ MSc.

Erin D Clarke,^1,2^ APD, PhD.

Jordan Stanford,^1,2^ APD, PhD.

Jessica J A Ferguson,^1,2^ APD, PhD.

Clare E Collins,^1,2^ FDA, PhD.

**Author Affiliations:**

1. School of Health Sciences, College of Health, Medicine and Wellbeing, The University of Newcastle, Callaghan, NSW, 2308 Australia
2. Food and Nutrition Research Program, Hunter Medical Research Institute, New Lambton Heights, NSW, 2305 Australia
3. Division of Human Nutrition and Health, Wageningen University and Research, Wageningen, 6702 PB, The Netherlands

**Corresponding author:**L/Prof Clare Collins

Advanced Technology Centre, Room 310, The University of Newcastle, University Drive, Callaghan, NSW 2308, Australia

Email: [clare.collins@newcastle.edu.au](mailto:clare.collins@newcastle.edu.au)

**Table S1.** Preferred Reporting Items for Systematic reviews and Meta-Analyses extension for Scoping Reviews (PRISMA-ScR) Checklist.

| **Section and Topic** | **Item #** | **Checklist item** | **Location where item is reported** |
| --- | --- | --- | --- |
| **TITLE** | | |  |
| Title | 1 | Identify the report as a systematic review. | 1 |
| **ABSTRACT** | | |  |
| Abstract | 2 | See the PRISMA 2020 for Abstracts checklist. | 2 |
| **INTRODUCTION** | | |  |
| Rationale | 3 | Describe the rationale for the review in the context of existing knowledge. | 3-4 |
| Objectives | 4 | Provide an explicit statement of the objective(s) or question(s) the review addresses. | 4 |
| **METHODS** | | |  |
| Eligibility criteria | 5 | Specify the inclusion and exclusion criteria for the review and how studies were grouped for the syntheses. | 5-6 |
| Information sources | 6 | Specify all databases, registers, websites, organisations, reference lists and other sources searched or consulted to identify studies. Specify the date when each source was last searched or consulted. | 5 |
| Search strategy | 7 | Present the full search strategies for all databases, registers and websites, including any filters and limits used. | 4-5 and Table S2 |
| Selection process | 8 | Specify the methods used to decide whether a study met the inclusion criteria of the review, including how many reviewers screened each record and each report retrieved, whether they worked independently, and if applicable, details of automation tools used in the process. | 5-6 |
| Data collection process | 9 | Specify the methods used to collect data from reports, including how many reviewers collected data from each report, whether they worked independently, any processes for obtaining or confirming data from study investigators, and if applicable, details of automation tools used in the process. | 5-6 |
| Data items | 10a | List and define all outcomes for which data were sought. Specify whether all results that were compatible with each outcome domain in each study were sought (e.g. for all measures, time points, analyses), and if not, the methods used to decide which results to collect. | 5-6 |
|  | 10b | List and define all other variables for which data were sought (e.g. participant and intervention characteristics, funding sources). Describe any assumptions made about any missing or unclear information. | 6 |
| Study risk of bias assessment | 11 | Specify the methods used to assess risk of bias in the included studies, including details of the tool(s) used, how many reviewers assessed each study and whether they worked independently, and if applicable, details of automation tools used in the process. | 6-7 |
| Effect measures | 12 | Specify for each outcome the effect measure(s) (e.g. risk ratio, mean difference) used in the synthesis or presentation of results. | 6 |
| Synthesis methods | 13a | Describe the processes used to decide which studies were eligible for each synthesis (e.g. tabulating the study intervention characteristics and comparing against the planned groups for each synthesis (item #5)). | 5-6 |
|  | 13b | Describe any methods required to prepare the data for presentation or synthesis, such as handling of missing summary statistics, or data conversions. | 6 |
|  | 13c | Describe any methods used to tabulate or visually display results of individual studies and syntheses. | 6 |
|  | 13d | Describe any methods used to synthesize results and provide a rationale for the choice(s). If meta-analysis was performed, describe the model(s), method(s) to identify the presence and extent of statistical heterogeneity, and software package(s) used. | 6 |
|  | 13e | Describe any methods used to explore possible causes of heterogeneity among study results (e.g. subgroup analysis, meta-regression). | 6 |
|  | 13f | Describe any sensitivity analyses conducted to assess robustness of the synthesized results. | NA |
| Reporting bias assessment | 14 | Describe any methods used to assess risk of bias due to missing results in a synthesis (arising from reporting biases). | 6-7 |
| Certainty assessment | 15 | Describe any methods used to assess certainty (or confidence) in the body of evidence for an outcome. | 6-7 |
| **RESULTS** | | |  |
| Study selection | 16a | Describe the results of the search and selection process, from the number of records identified in the search to the number of studies included in the review, ideally using a flow diagram. | 7-20 |
|  | 16b | Cite studies that might appear to meet the inclusion criteria, but which were excluded, and explain why they were excluded. | 7 |
| Study characteristics | 17 | Cite each included study and present its characteristics. | 7-9 |
| Risk of bias in studies | 18 | Present assessments of risk of bias for each included study. | 8 and Figure S1 |
| Results of individual studies | 19 | For all outcomes, present, for each study: (a) summary statistics for each group (where appropriate) and (b) an effect estimate and its precision (e.g. confidence/credible interval), ideally using structured tables or plots. | 7-20 |
| Results of syntheses | 20a | For each synthesis, briefly summarise the characteristics and risk of bias among contributing studies. | 8 |
|  | 20b | Present results of all statistical syntheses conducted. If meta-analysis was done, present for each the summary estimate and its precision (e.g. confidence/credible interval) and measures of statistical heterogeneity. If comparing groups, describe the direction of the effect. | 7-20 |
|  | 20c | Present results of all investigations of possible causes of heterogeneity among study results. | 7-20 |
|  | 20d | Present results of all sensitivity analyses conducted to assess the robustness of the synthesized results. | 7-20 |
| Reporting biases | 21 | Present assessments of risk of bias due to missing results (arising from reporting biases) for each synthesis assessed. | 7-20 |
| Certainty of evidence | 22 | Present assessments of certainty (or confidence) in the body of evidence for each outcome assessed. | NA |
| **DISCUSSION** | | |  |
| Discussion | 23a | Provide a general interpretation of the results in the context of other evidence. | 21 |
|  | 23b | Discuss any limitations of the evidence included in the review. | 24-25 |
|  | 23c | Discuss any limitations of the review processes used. | 24-25 |
|  | 23d | Discuss implications of the results for practice, policy, and future research. | 25 |
| **OTHER INFORMATION** | | |  |
| Registration and protocol | 24a | Provide registration information for the review, including register name and registration number, or state that the review was not registered. | 5 |
|  | 24b | Indicate where the review protocol can be accessed, or state that a protocol was not prepared. | 5 |
|  | 24c | Describe and explain any amendments to information provided at registration or in the protocol. | 5 |
| Support | 25 | Describe sources of financial or non-financial support for the review, and the role of the funders or sponsors in the review. | 26 |
| Competing interests | 26 | Declare any competing interests of review authors. | 26 |
| Availability of data, code and other materials | 27 | Report which of the following are publicly available and where they can be found: template data collection forms; data extracted from included studies; data used for all analyses; analytic code; any other materials used in the review. | 26 |

*From:*  Page MJ, McKenzie JE, Bossuyt PM, Boutron I, Hoffmann TC, Mulrow CD, et al. The PRISMA 2020 statement: an updated guideline for reporting systematic reviews. BMJ 2021;372:n71. doi: 10.1136/bmj.n71

**Table S2.** Database search strategy.

| **Embase (Ovid)** |
| --- |
| (metabolome.tw. OR Metabolites.tw. OR metabolomics.tw. OR exp metabolomics/) AND (diet.tw. OR exp diet/ OR dietary.tw. OR "feeding study".tw.) AND ("blood pressure".tw. OR hypertension.tw. OR systolic.tw. OR diastolic.tw.) AND (intervention.tw. OR trial.tw.) + (full text and human and english language and yr="2024") |
| **Medline (Ovid)** |
| ( TITLE-ABS ( metabolome ) OR TITLE-ABS ( metabolites ) OR TITLE-ABS ( metabolomics ) OR INDEXTERMS ( metabolomics ) ) AND ( TITLE-ABS ( diet ) OR INDEXTERMS ( diet ) OR TITLE-ABS ( dietary ) OR TITLE-ABS ( "feeding study" ) ) AND ( TITLE-ABS ( "blood pressure" ) OR TITLE-ABS ( hypertension ) OR TITLE-ABS ( systolic ) OR TITLE-ABS ( diastolic ) ) AND ( TITLE-ABS ( intervention ) OR TITLE-ABS ( trial ) ) AND ( LIMIT-TO ( PUBYEAR , 2023 ) ) AND ( LIMIT-TO ( DOCTYPE , "ar" ) ) AND ( LIMIT-TO ( LANGUAGE , "English" ) ) AND ( LIMIT-TO ( EXACTKEYWORD , "Human" ) OR LIMIT-TO ( EXACTKEYWORD , "Humans" ) ) AND ( LIMIT-TO ( SRCTYPE , "j" ) ) (english language and humans and yr="2024" |
| **Scopus** |
| ( metabolome OR metabolites OR metabolomics OR metabolomics ) AND ( diet OR diet OR dietary OR "feeding study" ) AND ( "blood pressure" OR hypertension OR systolic OR diastolic ) AND ( intervention OR trial ) AND ( LIMIT-TO ( DOCTYPE , "ar" ) ) AND ( LIMIT-TO ( LANGUAGE , "English" ) ) AND ( LIMIT-TO ( EXACTKEYWORD , "Human" ) OR LIMIT-TO ( EXACTKEYWORD , "Humans" ) ) AND ( LIMIT-TO ( PUBYEAR , 2024 ) ) AND ( LIMIT-TO ( SRCTYPE , "j" ) )  Scopus advances search  ( TITLE-ABS ( metabolome ) OR TITLE-ABS ( metabolites ) OR TITLE-ABS ( metabolomics ) OR INDEXTERMS ( metabolomics ) ) AND ( TITLE-ABS ( diet ) OR INDEXTERMS ( diet ) OR TITLE-ABS ( dietary ) OR TITLE-ABS ( "feeding study" ) ) AND ( TITLE-ABS ( "blood pressure" ) OR TITLE-ABS ( hypertension ) OR TITLE-ABS ( systolic ) OR TITLE-ABS ( diastolic ) ) AND ( TITLE-ABS ( intervention ) OR TITLE-ABS ( trial ) ) AND ( LIMIT-TO ( PUBYEAR , 2024 ) ) AND ( LIMIT-TO ( DOCTYPE , "ar" ) ) AND ( LIMIT-TO ( LANGUAGE , "English" ) ) AND ( LIMIT-TO ( EXACTKEYWORD , "Human" ) OR LIMIT-TO ( EXACTKEYWORD , "Humans" ) ) AND ( LIMIT-TO ( SRCTYPE , "j" ) ) |
| **CINAHL** |
| ((TI metabolome OR AB metabolome) OR (TI Metabolites OR AB Metabolites) OR (TI metabolomics OR AB metabolomics) OR (MH metabolomics+)) AND ((TI diet OR AB diet) OR (MH diet+) OR (TI dietary OR AB dietary) OR (TI "feeding study" OR AB "feeding study")) AND ((TI "blood pressure" OR AB "blood pressure") OR (TI hypertension OR AB hypertension) OR (TI systolic OR AB systolic) OR (TI diastolic OR AB diastolic)) AND ((TI intervention OR AB intervention) OR (TI trial OR AB trial))  Limiters - Full Text; Published Date: 20241211; English Language; Human |
| **Cochrane** |
| (metabolome:ti,ab OR Metabolites:ti,ab OR metabolomics:ti,ab OR [mh metabolomics]) AND (diet:ti,ab OR [mh diet] OR dietary:ti,ab OR “feeding study”:ti,ab) AND (“blood pressure”:ti,ab OR hypertension:ti,ab OR systolic:ti,ab OR diastolic:ti,ab) AND (intervention:ti,ab OR trial:ti,ab)” |
| **PsynchINFO** |
| (metabolome.ti,ab. OR Metabolites.ti,ab. OR metabolomics.ti,ab. OR exp metabolomics/) AND (diet.ti,ab. OR exp diet/ OR dietary.ti,ab. OR "feeding study".ti,ab.) AND ("blood pressure".ti,ab. OR hypertension.ti,ab. OR systolic.ti,ab. OR diastolic.ti,ab.) AND (intervention.ti,ab. OR trial.ti,ab.) + English, humans, 2024. Full text |

| **Author** | **Validity Questions** | | | | | | | | | | **Overall quality score** |
| --- | --- | --- | --- | --- | --- | --- | --- | --- | --- | --- | --- |
|  | **1** | **2** | **3** | **4** | **5** | **6** | **7** | **8** | **9** | **10** |  |
| Cheng, 2018 |  |  |  |  |  |  |  |  |  |  | Neutral |
| Chen, 2019 |  |  |  |  |  |  |  |  |  |  | Positive |
| Huang, 2021 |  |  |  |  |  |  |  |  |  |  | Positive |
| Reisdorph, 2020 |  |  |  |  |  |  |  |  |  |  | Positive |
| Loo, 2018 |  |  |  |  |  |  |  |  |  |  | Positive |
| Han, 2022 |  |  |  |  |  |  |  |  |  |  | Positive |
| Kim, 2023 |  |  |  |  |  |  |  |  |  |  | Neutral |
| Trimigno, 2023 |  |  |  |  |  |  |  |  |  |  | Positive |
| Qadir, 2022 |  |  |  |  |  |  |  |  |  |  | Positive |
| Sallem, 2023 |  |  |  |  |  |  |  |  |  |  | Positive |
| Changwei, 2023 |  |  |  |  |  |  |  |  |  |  | Positive |
| Sun, 2024 |  |  |  |  |  |  |  |  |  |  | Neutral |

**Fig. S1** American Dietetic Association (ADA) Quality Criteria Checklist carried out on the 10 included articles

(1) Clear Research Question; (2) Unbiased Selection of Participants; (3) Randomization/Group Comparability; (4) Description of Withdrawals; (5) Blinding; (6) Study Procedures Described; (7) Clearly Defined Outcomes; (8) Appropriate Statistical Analysis; (9) Results Support Conclusion; (10) Funding or Sponsorship Bias Unlikely. To be rated positive, each of criteria 2, 3, 6 and 7 must be met and the majority of 10 criteria overall. Any of criteria 2, 3, 6 and 7 not being met resulted in a neutral rating. The majority of criteria not being met results in a negative rating. Positive, negative and unsure answers were represented by colours green, red and oranges respectively.

**Table S3.** Summary of included studies.

| ***Ref, Year*** | ***Location. Study Design*** | ***Population Characteristics*** | | | | | | | | | | | | |
| --- | --- | --- | --- | --- | --- | --- | --- | --- | --- | --- | --- | --- | --- | --- |
|  |  | ***Age (y)*** (mean±SD) | ***Pop.***  ***N (%M)*** | ***Pop.***  ***Condition*** | ***Run-in period*** | ***Length of each feeding intervention*** | ***Length total intervention*** | ***Washout period*** | ***Total length*** | ***Dietary Patterns*** | ***Bio-samples*** | ***Method used to assess metabolome*** | ***Targeted***  ***Vs***  ***Un-targeted Analysis*** | ***Blood Pressure office/ambulant*** |
| **Cheng, 2018** | USA.  Randomised (parallel and cross-over design). | 49±10 | 103 (42) | Adults with  higher than optimal blood pressure or Stage 1  hypertension (SBP, 120-159 mmHg; DBP, 80-95  mmHg) | Y, 2 w | 30 d | 60 d | N | 74 d (≈11 w) | DASH-Sodium trial, but only the participants in the Typical US diet.  **INT**: low sodium (50 mmol/day)  **INT:** high sodium (150mmol/day)  **Salt-sensitivity**: >10 mmHg (SBP and DBP) on high-sodium vs. low-sodium diet. **Salt-insensitive:** 5 mmHg or less on high-sodium vs. low-sodium diet. | Urine  Blood | UPLC-MS and GC-MS (urine) | Targeted | Unclear (Office/ambulatory)  According to ref.14 and 15 of the article:  (DASH (ref 14) measured in sitting position, using random-zero sphygmomanometers, at different times.  Screening: 3  Run-in Period: twice.  Intervention: Weekly during the first 3 weeks of each 30-day intervention period.  Visits: at 5 clinic visits during the last 9 days of each intervention period.  DASH sodium trial (ref 15), BP was recorded using random-zero sphygmomanometer, with 2 readings taken per visit (5 minutes of quiet sitting before the first, and 30 seconds after the first). Baseline BP was the average of 3 pairs of screening and 2 run-in measurements. End-of-intervention BP is the mean of 5 pairs of measurements during the last 9 days of the intervention period (at least 2 during the final 4 days). A 24-hour ambulatory BP measurement was also taken during the last 9 days using a SpaceLabs 90207 device. |
| **Loo, 2018** | USA  Randomised, controlled cross-over feeding study. | 53.1±10.8 | 158 (56) | Adults with prehypertension (SBP of  120–139 mmHg and/or DBP of 80–89 mmHg) or stage  1 hypertension (SBP of 140–159 mm Hg and/or DBP of 90–99 mm Hg) and without diabetes or prior CVD | Y, 6 d | 6 w | 18 w | Y, ≥ 2 w | ≥ 22 w | **INT**:  **OmniProt:** 48% kcal from carbohydrate, 25% from protein, and 27% from fat  **INT:**  **OmniCarb:** 58% kcal from carbohydrate,15% from protein, and 27% from fat  **INT**:  **OmniMFA:** 48% kcal from carbohydrate, 15% from protein, and 37% from fat | Urine | ^1^H-NMR | Targeted | Office  (BP was measured on 5 d using the OMRON 907 device. Participants seated for ≥5 minutes and based on the average of 9 measurements at screening visits and 15 in the last 5 visits of each feeding period) |
| **Chen, 2019** | UK. Randomised, double-blind, placebo-controlled cross-over trial. | 50.2±9.5 | 64 (50) | Untreated black adults’ hypertensives  (SBP 140 to 170 mm Hg or DBP 90 to 105 mm Hg) | Y, 2 w | 6 w | 12 w | N | 14 w | **INT**: reduced-sodium diet + sodium tablets.  **INT**: reduced-sodium diet + placebo | Serum,  Urine | UPLC-MS/MS (serum) | Untargeted | 24h ambulatory BP/office  Office  (After each intervention after participants were seated for ≥5 minutes, using a digital  BP monitor (Omron HEM-705CP). Three readings were taken, and the average of the last 2 readings was used)  Ambulatory  Was measured performed using SpaceLabs 90207 devices |
| **Reisdorph, 2020** | USA.  Randomised, cross-over, controlled feeding study | 61±2 | 19 (32) | Adults prehypertensive  Inclusion criteria: SBP ≥120 mm Hg or DBP ≥80 mm Hg. Sample was prehypertensive SBP 130 mm Hg and DBP 85 mm Hg) | Y, 2 w | 6 w | 12 w | Y,4 w | 18 w | **INT:** DASH-style diet with lean pork  **INT:** DASH-style diet with chicken/fish | Urine | HPLC-MS | Untargeted | 24h ambulant  (BP measured for 3 consecutive days during each of the four measurement periods monitoring using a 24-h ambulatory BP  monitoring system (SunTech Medical, Inc., Morrisville, NC, USA)) |
| **Huang, 2021** | USA.  Randomised, controlled, double-blinded, 2-arm, 2-period crossover trial. | 53±1 | 34 (50) | Adults ( 40–69 y) overweight/obese with moderate hypercholesterolemia | N | 4 w | 8 w | Y,4 w | 12 w | **INT:** beverages containing freeze-dried strawberry powder  **CON:** beverages containing energy-, volume-matched control (placebo) | Plasma | UHPLC-ESI-MS/MS | Targeted | Office  (BP was measured in sitting position (fasting state) and repeated 3 times at 5-min intervals at  0 h, before the 1-h flow-mediated dilation measurement, and at 2 h, using FDA-approved equipment) |
| **Han, 2022** | China.  Single-arm intervention study. | 60-70* | 14 (50) | Adults with CVD risk and not taking antibiotics, probiotics/prebiotics or vitamins or medication that affect the immune system | Y, ≥ 2 w | 2 w | 2 w | N | ≥ 4 w | **INT:** dietary pattern derived from centenarians Guangxi, China | Faeces, plasma | ^1^H-NMR (faeces) | Untargeted | Office  (BP was measured using a sphygmomanometer) |
| **Qadir, 2022** | UK.  Cross-over, with randomised, double-blind. | 25.0±2.9 | 19 (42.8) | Healthy adults | N | 1 d | 2 d | Y, 3 w | ≈ 3 w | **Intervention: Lettuce (50g) with** high NO_3_^−^ (530 mg) and low phenolics contents (11 mg) (HNLP)  **Intervention: Lettuce with (50g)** low NO_3_^−^ (3 mg) and high phenolics (77 mg) (LNHP) | Plasma, urine and saliva | GC–MS for NO_3_^−^ (plasma, saliva and urine), and NO_2_^−^ (saliva)  UHPLC-ESI-QqQ-MS/MS for plasma phenolic compounds | Targeted | 24h ambulatory  (Baseline blood pressure was measured three times with a Mobil-O-Graph NG, and the device was then used to monitor BP for 24 hours, with measurements taken every 30 minutes during the day and every 60 minutes at night) |
| **Kim, 2023** | USA  2 randomised controlled feeding studies. The DASH (parallel arm design) and DASH-Sodium The DASH,  comparing  the effects of 3 levels of sodium intake and 2 dietary patterns (parallel arm design).  Within each  diet intervention of the DASH-Sodium trial, participants received high, intermediate, and low sodium in random order for 4 weeks  (crossover design). | > 30 DASH trial 88.1% and DASH sodium 96.7%* | DASH 218 (52.3) DASH sodium 395 (43.5) | Adults (≥22 y) with higher than optimal BP or with stage 1 hypertension  DASH trial: with SBP <160 mm Hg and DBP of 80 to 95 mm Hg  DASH sodium: with SBP of 120 to 159 mm Hg and DBP of  80 to 95 mm Hg. | Y, 3 w (DASH trial) and 2 w (DASH-sodium) | **DASH trial:** 8 w  **DASH sodium**: 4 w | ≈ 12 w | N | **DASH trial**: 11 w  **DASH sodium trial:** 14 w | ***DASH trial***  **INT**: DASH diet and fruit and vegetable diet  **CON:** Control diet (typical American diet).  ***DASH sodium***  **INT:** DASH high-sodium  **CON:** control high-sodium diet (DASH-sodium trial)  **INT:** DASH low-sodium  **CON:** control high-sodium diet (DASH-sodium trial) | Urine (DASH sodium trial) and serum (DASH- trial) | UPLC-MS/MS | Untargeted | Office  (BP measurements were obtained during screening visits, run-in visits, and  intervention periods. In both trials, 2 blood pressure measurements  were obtained after a 5-minute period of sitting, using a random-zero sphygmomanometer.) |
| **Sellem, 2023** | UK.  Single-blind, parallel RCT | 43.8±10.2 (DIVAS) | 113 (39.8) | Adults with a moderate CVD risk (DIVAS)  EPIC-Potsdam T2D and CVD risks | N | 16 w | 16 w | N | 16 w | **INT:** SFA-rich diet (SFA:MUFA:n-6 PUFA ratio=17:11:4 % TE)  **INT:** MUFA-rich diet (SFA:MUFA:n-6 PUFA ratio=9:19:4 % TE)  **INT:**MUFA/PUFA-rich diet (target SFA:MUFA:n-6 PUFA ratio=9:13:10% TE) | Plasma | CLP | Targeted | 24h ambulatory  According to Ref. 14 of the article:  BP was measured using an A/A grade automated oscillometric  monitors (A&D Instruments Ltd.), and was measured every 30 min during the day and  every 60 during the night, approximately 48 h before the  clinical visits. Mean 24-h day and night measurements were  calculated by using sleep times recorded on participant activity  forms. |
| **Trimigno, 2023** | Denmark. Randomised, parallel, and controlled dietary intervention | NDD:  44.15 ± 13.1  ADD:  40.8 ± 13.2 | 146 (31.5)  NND; 90 (33)  ADD: 56 (29) | Centrally obese adults | Y, 1 w | 26 w | 26 w | N | 27 w | **INT:** New Nordic Diet (NND): higher content of organic food including whole grains, nuts, fruit and vegetables, fish and seafood, and lower of meat  **CONT:** Average Danish Diet (ADD), high in refined grains meat, dairy and cheese, sugar and convenience foods and low-fiber vegetables and fruit | Plasma | ^1^H-NMR | Untargeted | Office |
| **Changwei, 2023** | USA. Randomized controlled, crossover  trial of dietary interventions | ProBP |  | Adults with prehypertension or stage-1 hypertension not taking antihypertensive mediation  SBP between 120 and 159  mmHg and a DBP between 80 and 95 mmHg and not  taking antihypertensive mediation | Y, 2 w | 8 w | 24 w | Y, 3 w | 32 w | **INT:** ProBP study ProBP-soy, 40 g/d of soy protein supplement  **INT**: ProBP-milk, 40 g/d of milk protein supplement **INT**: ProBP-carbs, 40 g/d of carbohydrates supplement | Serum | UPLC-MS/MS | Untargeted | Office  (BP was measured twice at the beginning and twice at the end of an intervention phase. During each examination, BP was measured 3 times  using the Hawksley random-zero sphygmomanometer) |
| **Sun, 2024** |  | 47.6 ± 10.1 | 55 (68.7) | Adults with prehypertension or stage-1 hypertension not taking antihypertensive mediation | Y, 2 w | 8 w | 24 w | Y, 3 w | 32 w | **INT:** ProBP study ProBP-soy, 40 g/d of soy protein supplement  **INT**: ProBP-milk, 40 g/d of milk protein supplement **INT**: ProBP-carbs, 40 g/d of carbohydrates supplement | Plasma | UPLC-MS/MS | Untargeted | Office  (BP was measured twice at the beginning and twice at the end of an intervention phase. During each examination, BP was measured 3 times  using the Hawksley random-zero sphygmomanometer) |

BP, blood pressure. CVD, cardiovascular disease. CLP, Complex Lipids Platform™. GC-MS, Gas chromatography- mass spectrometry. D, days. H-NMR, Proton nuclear magnetic resonance. HPLC-MS, high performance liquid chromatography- mass spectrometry. M, month. R, reference. Pop, population. UHPLC-ESI-MS/MS, ultra-high performance liquid chromatography–electrospray ionization tandem mass spectrometry. UHPLC-ESI-QqQ-MS/MS Ultra High-Performance Liquid Chromatography Electrospray Ionization Triple Quadrupole Mass Spectrometry. UK, United Kingdom. UPLC-MS: Ultra high-performance liquid chromatography- mass spectrometry. USA, United States of America. W, weeks. * range of years.

**Table S4.** Summary of the results found in the articles included in this review.

|  |  |  | ***Results*** | | | |
| --- | --- | --- | --- | --- | --- | --- |
| ***REF*** | ***Biofluid*** | ***Diet*** | ***Difference in BP after intervention*** | ***Metabolites measured*** | ***Was the relationship between metabolite and BP change measured?*** | ***Relationship between BP and metabolites p-value association*** |
| **Cheng, 2018** | Urine | INT: low sodium  INT: high sodium | Unclear  Salt-sensitive  SBP:  Low sodium: 123 ± 8 mmHg  High sodium: 140 ± 6 mmHg  DBP:  Low sodium: 78 ± 9 mmHg  High sodium: 87 ± 6 mmHg  Salt-insensitive  Very similar between low- and high-sodium intakes (123 ± 7 mmHg and 80 ± 5  mmHg, respectively, for SBP and DBP) | 47 amino metabolites were measured and 10 tricarboxylic acid metabolites.  5 metabolites were associated with BP. | Unclear | Association between metabolites and BP:  **SBP**: (no ≠between diets)   \| β-aminoisobutyric acid \| 0.026 \| - \| \| --- \| --- \| --- \| \| Cystine \| 0.008 \| + \| \| Citrulline \| 0.003 \| + \| \| Homocysteine \| 0.025 \| + \| \| Lysine \| 0.009 \| + \|   **DBP**   \| Cystine \| 0.005 \| + \| \| --- \| --- \| --- \|   When metabolite and DBP differences between low- and high-sodium intakes were analysed, homocysteine was the only metabolite significantly associated with DBP.  None of the metabolites showed significant association with SBP at the level of FDR<0.05 when differences between low- and high-sodium intakes were analysed by GLM |
| **Loo, 2018** | Urine | OmniDiet  High in carbohydrates, protein or monounsaturated fatty acids | SBP^§^  OmniCarb -8.0 (-9.4, -6.6)  OmniMFA -9.4 (-10.7, -8.1)  OmniProt -9.4 (-10.8, -8.1) *P*<10^−10^ for all  DBP  OmniCarb -4.1 (-4.9, -3.3)  OmniMFA -4.9 (-5.7, -4.1)  OmniProt -5.3 (-6.1, -4.4)  *P*<10^−10^ for all | Quantified 10 discriminatory metabolites altered in response to 1 or more OmniHeart diets and assessed their associations with BP.  BP was significantly associated with 6 metabolites (2 food-related metabolites (proline-betaine and carnitine), 3 metabolites related to host-gut microbial pathways (hippurate, phenylacetylglutamine, and 4-cresyl sulfate) and a tryptophan–NAD metabolite (*N*-methyl-2-pyridone-5-  Carboxamide) | Yes | Associations between metabolites and change in BP:  **SBP**   \| **OmniCarb vs. baseline** \|  \|  \| \| --- \| --- \| --- \| \| Proline-betaine \| <0.01 \| - \| \| Hippurate \| <0.001 \| + \| \| N-methyl-2-pyridone-5-carboxamide \| <0.05 \| - \| \| **OmniMFA vs. baseline** \|  \|  \| \| Proline-betaine \| <0.01 \| - \| \| **OmniProt vs. baseline** \|  \|  \| \| Carnitine \| <0.05 \| + \| \| **OmniMFA vs. OmniProt** \|  \|  \| \| 4-cresyl sulfate \| <0.01 \| - \|   **DBP**   \| **OmniCarb vs. baseline** \|  \|  \| \| --- \| --- \| --- \| \| Proline-betaine \| <0.05 \| - \| \| Hippurate \| <0.01 \| + \| \| N-methyl-2-pyridone-5-carboxamide \| <0.05 \| - \| \| **OmniMFA vs. baseline** \|  \|  \| \| Proline-betaine \| <0.05 \| - \| \| **OmniMFA vs. OmniProt** \|  \|  \| \| 4-cresyl sulfate \| <0.01 \| - \| \| Phenylacetylglutamine \| <0.01 \| - \| |
| **Chen, 2019** | Serum | Reduced-sodium diet + placebo | SBP:  Office:  Sodium reduction+placebo: 143.7±11.8 mmHg  Sodium reduction+sodium: 148.9±13.5mmHg  *P*<0.001  Ambulatory  24-hour:  Sodium reduction+placebo : 140.1±10.1 mmHg  Sodium reduction+sodium: 144.5±8.4 mmHg  *P*<0.001  Day  Sodium reduction+placebo: 144.9±10.4 mmHg  Sodium reduction+sodium: 150.1±9.3 mmHg  *P*<0.001  Night  Sodium reduction+placebo: 134.1±12.1 mmHg  Sodium reduction+sodium: 138.2±9.7 mmHg  *P*<0.001  DBP  Office:  Sodium reduction+placebo: 88.2 ± 9.1 mmHg  Sodium reduction+sodium: 90.7 ± 9.1 mmHg *P*<0.001  Ambulatory  24-hour:  Sodium reduction+placebo: 85.4 ± 9.0 mmHg  Sodium reduction+sodium: 87.8 ± 8.9 mmHg  *P*<0.001  Day  Sodium reduction+placebo; 89.9 ± 9.6 mmHg  Sodium reduction+sodium: 92.7 ± 9.7 mmHg  *P*<0.001  Night  Sodium reduction+placebo; 80.4 ± 9.8 mmHg  Sodium reduction+sodium: 82.4 ± 9.3 mmHg  *P*<0.001 | 870 metabolites were detected, 595 identified metabolites were entered to analysis.  34 metabolites responded with sodium reduction (*P*<0.05), of which 6 were upregulated by sodium reduction, 2 metabolites HMB (β-hydroxyisovalerate  β-hydroxy-β-methylbutyric acid) and methionine sulfone  remained significant after FDR correction. | Yes | Association between change in metabolites with change in BP:  **SBP:**  Office:   \| β-Hydroxyisovalerate β-hydroxy-β-methylbutyric acid \| <0.05 \| - \| \| --- \| --- \| --- \| \| Campesterol \| <0.01 \| + \| \| N2-Acetyllysine \| <0.01 \| - \| \| Sucrose \| <0.05 \| + \|     Ambulant  24-hours   \| N-Acetylneuraminate \| <0.05 \| - \| \| --- \| --- \| --- \| \| Sucrose \| <0.05 \| + \|   Day   \| β-Hydroxyisovalerate \| <0.05 \| - \| \| --- \| --- \| --- \| \| N-Acetylneuraminate \| <0.05 \| - \| \| Sucrose \| <0.05 \| + \|   Night   \| Sucrose \| <0.05 \| + \| \| --- \| --- \| --- \|   **DBP:**  Office:   \| N-Acetylneuraminate \| <0.05 \| - \| \| --- \| --- \| --- \| \| Campesterol \| <0.01 \| + \| \| N2-Acetyllysine \| <0.01 \| - \|   Ambulant:  24-hours   \| Methionine Sulfone \| <0.05 \| - \| \| --- \| --- \| --- \| \| N-Acetylneuraminate \| <0.01 \| - \| \| Campesterol \| <0.05 \| + \| \| Sucrose \| <0.05 \| + \| \| Vanillactate \| <0.05 \| - \|   Day   \| N-Acetylneuraminate \| <0.01 \| - \| \| --- \| --- \| --- \| \| Campesterol \| <0.05 \| + \|   Night   \| Methionine Sulfone \| <0.05 \| - \| \| --- \| --- \| --- \| \| N-Acetylneuraminate \| <0.01 \| - \| \| Sucrose \| <0.01 \| + \| |
| **Reisdorph, 2020** | Urine | DASH-diet | NA | 1,960 compounds were detected in both pre-diet and post-diet urine. 90 compounds were analysed.  16 metabolites were associated with BP and 6 metabolites with changes in BP | Both  1-Associated with BP  2-Associated with BP changes | Associated with BP  **SBP**   \| 2-Acetyl-3-methylpyrazine \| <0.01 \|  \| \| --- \| --- \| --- \| \| 2-(3-Methylthiopropyl)malate \| <0.05 \|  \| \| L-Glutamic acid \| <0.05 \|  \| \| VAL-GLU \| <0.01 \|  \| \| N-(Phenylacetyl)glutamic Acid \| <0.05 \|  \| \| 73.0264 \| <0.05 \|  \| \| 121.917 \| <0.001 \|  \| \| 124.039 \| <0.01 \|  \| \| 238.1336 \| <0.05 \|  \| \| 268.1409 \| <0.001 \|  \|   **DBP**   \| 3-(3-Methylbutylidene)-1(3 H)-isobenzofuranone \| <0.05 \| \| --- \| --- \| \| 2-Acetyl-3-methylpyrazine \| <0.01 \| \| 3-Indolebutyric acid \| <0.05 \| \| 2-(3-Methylthiopropyl)malate \| <0.05 \| \| Bicine \| <0.05 \| \| L-Glutamic acid \| <0.05 \| \| N-Acetylneuraminic acid \| <0.05 \| \| Potassium gluconate \| <0.05 \| \| VAL-GLU \| <0.05 \| \| 121.917 \| <0.001 \| \| 124.039 \| <0.01 \| \| 238.1336 \| <0.05 \| \| 268.1409 \| <0.001 \| \| 291.0951 \| <0.05 \|   Associations between metabolites and change in BP:  **SBP**   \| 3-Indolebutyric acid \| <0.01 \| - \| \| --- \| --- \| --- \| \| 1-(beta-D-Ribofuranosyl)−1,4-dihydronicotinamide \| <0.05 \| + \| \| Kynuramine \| <0.05 \| - \| \| Physoperuvine \| <0.05 \| - \| \| 265.0971 \| <0.05 \| - \| \| 157.0373 \| <0.05 \| - \|   **DBP**   \| 3-Indolebutyric acid \| <0.05 \| - \| \| --- \| --- \| --- \| \| Physoperuvine \| <0.05 \| - \| |
| **Huang, 2021** | Plasma | Strawberries beverage | SBP Strawberry: 120 ± 2 mmHg; Control: 119 ± 2 mmHg *P*=1.00  DBP:  Strawberry: 81 ± 2 mmHg: Control: 81 ± 2 mmHg. *P*=1.00 | In total 142 metabolites were quantified, of which 17 significantly changed and were analysed further.  No correlation was found between metabolites and BP. | Yes | No significant correlation between metabolites and change in BP. |
| **Han, 2022** | Faeces | Derived from centenarians Guangxi | SBP:  Pre: 132.50 ± 20.47 mmHg  Post: 122.50 ± 11.22 mmHg  *P*=0.041  DBP:  Pre: 79.93 ± 11.84 mmHg  Post: 76.64 ± 9.83 mmHg  *P*=0.044 | 45 different metabolites were identified and quantified.  Nine metabolites showed significant changes compared with pre-intervention. 2 were increased and 7 decreased.  2 metabolites were associated with BP | Unclear | Correlation between faecal metabolites and BP  **SBP:**   \| Alanine \| <0.05 \| + \| \| --- \| --- \| --- \| \| Aspartate \| <0.01 \| + \|   **DBP:**   \| Aspartate \| <0.05 \| + \| \| --- \| --- \| --- \| |
| **Qadir, 2022** | Plasma, urine and saliva | **Intervention: Lettuce with** high NO_3_^−^ and low phenolics contents (HNLP)  **Intervention: Lettuce with** low NO3^−^ and high phenolics (LNHP) | SBP:  After 6 hours, LNHP higher than HNLP (*P*=0.031) | Inorganic nitrate (NO_3_^−^) and nitrite (NO_2_^−^) and phenolic compounds | Yes | HNLP  **SBP**   \| NO_3_^−^ (saliva) \| <0.01 \| - \| \| --- \| --- \| --- \| |
| **Kim, 2023** | Urine (DASH sodium trial) and plasma (DASH-sodium trial) | ***DASH trial***  **INT**: DASH. Fruit and vegetable diet  **CON:** Control diet  ***DASH sodium***  **INT:** DASH high-sodium  **CON:** control high-sodium diet (DASH-sodium trial)  **INT:** DASH low-sodium  **CON:** control high-sodium diet (DASH-sodium trial) | ***NA*** | Of the 65 interactions, 42 unique metabolites were associated with either SBP or DBP (9 serum metabolites and 33 urine) | Yes | Association between metabolites and change in BP:  ***DASH trial: (serum metabolites)***  ***DASH-SBP***   \| Glycosyl-N-stearoyl-sphingosine (d18:1/18:0) \| 0.02 \| + \| \| --- \| --- \| --- \| \| Carotene diol (3) \| 0.04 \| + \| \| Glycosyl-N-palmitoyl-sphingosine (d18:1/16:0) \| 0.05 \| + \| \| 1-palmitoyl-2-oleoyl-GPC (16:0/18:1) \| <0.01 \| + \| \| 1-stearoyl-2-oleoyl-GPC (18:0/18:1) \| <0.01 \| + \|   ***Control-SBP***   \| Glycosyl-N-stearoyl-sphingosine (d18:1/18:0) \| 0.03 \| - \| \| --- \| --- \| --- \|   ***DASH-DPB***   \| Tryptophan betaine \| <0.01 \| - \| \| --- \| --- \| --- \| \| Glycosyl-N-palmitoyl-sphingosine \| 0.02 \| + \| \| 1-palmitoyl-2-oleoyl-GPC (16:0/18:1) \| <0.01 \| + \|   ***DASH -High Sodium trial: (urine metabolites)***  ***DASH -High Sodium compared to control-high sodium***  ***SBP***   \| N-carbamoylvaline \| 0.04 \| - \| \| --- \| --- \| --- \| \| N-methylhydroxyproline \| <0.01 \| - \| \| Dopamine 3-O-sulfate \| 0.02 \| - \| \| N,N-dimethyl-5-aminovalerate \| 0.05 \| - \| \| Alpha-CEHC taurine \| <0.01 \| - \| \| Pantothenate (Vitamin B5) \| <0.01 \| - \| \| Pyridoxate \| <0.01 \| - \| \| Alpha-CEHC glucuronide \| 0.02 \| - \| \| Picolinoylglycine \| 0.01 \| - \| \| Allantoin \| 0.02 \| - \| \| Phenylacetylglycine \| <0.01 \| - \| \| 3-hydroxyphenylacetoylglutamine \| 0.05 \| - \| \| Stachydrine \| 0.03 \| - \|   ***Control high-sodium diet SBP***   \| N-carbamoylvaline \| <0.01 \| + \| \| --- \| --- \| --- \| \| 3,4-dihydroxyphenylacetate sulfate \| <0.05 \| + \| \| 4-hydroxycinnamate \| 0.03 \| + \| \| N-acetylalliin \| 0.02 \| + \|   ***DASH -High Sodium***  ***DBP***   \| 3,4-dihydroxyphenylacetate sulfate \| <0.01 \| - \| \| --- \| --- \| --- \| \| Dopamine 4-sulfate \| 0.01 \| - \| \| N-methylproline \| 0.02 \| - \| \| Dopamine 3-O-sulfate \| <0.01 \| - \| \| Thiamine (Vitamin B1) \| <0.01 \| - \| \| Pantothenate (Vitamin B5) \| <0.01 \| - \| \| Riboflavin (Vitamin B2) \| <0.01 \| - \| \| Phenylacetylglycine \| <0.01 \| - \| \| 4-hydroxymandelate \| 0.01 \| - \| \| 2,8-quinolinediol sulfate \| <0.01 \| - \| \| Stachydrine \| <0.01 \| - \|   ***Control high-sodium diet DBP***   \| 3,4-dihydroxyphenylacetate sulfate \| 0.02 \| + \| \| --- \| --- \| --- \| \| 3-hydroxyindolin-2-one \| <0.05 \| + \| \| 4-hydroxycinnamate \| <0.01 \| + \|   ***DASH-low sodium compared to control-high sodium***  ***SBP***   \| N-methylglutamate \| 0.02 \| - \| \| --- \| --- \| --- \| \| 3,4-dihydroxyphenylacetate sulfate \| 0.01 \| - \| \| N-methylhydroxyproline \| 0.04 \| - \| \| alpha-CEHC taurine \| 0.02 \| - \| \| alpha-CEHC glucuronide \| <0.01 \| - \| \| 3- hydroxyphenylacetoylglutamine \| <0.01 \| - \| \| Stachydrine \| <0.01 \| - \| \| 3-hydroxystachydrine \| 0.02 \| - \|   ***Control-high sodium* SBP**   \| N,N-dimethylalanine \| 0.01 \| - \| \| --- \| --- \| --- \| \| 3,4-dihydroxyphenylacetate sulfate \| 0.05 \| + \| \| gamma-glutamyl-alpha- lysine \| 0.04 \| - \|   ***DASH-low sodium compared to control-high sodium DBP***   \| Alpha-CEHC glucuronide \| <0.01 \| - \| \| --- \| --- \| --- \| \| Pantothenate (Vitamin B5) \| <0.01 \| - \|   ***Control-high sodium DBP***   \| 3,4-dihydroxyphenylacetate sulfate \| 0.02 \| + \| \| --- \| --- \| --- \| \| 3-(4-hydroxyphenyl)lactate \| 0.05 \| + \| \| N-acetylalliin \| 0.02 \| + \|   *All showed P values <0.050 between intervention. In addition,*  *tryptophan betaine, 2-hydroxysebacate, abscisate, carnosine, 5-hydroxymethyl-2-furoic acid showed P values <0.050 between intervention.* |
| **Sellem, 2023** | Plasma | **INT:** SFA-rich diet   **INT:** MUFA-rich diet  **INT:** MUFA/PUFA-rich diet | NA | 987 were identified and 886 molecular species were retained. 28 different fatty acids were detected and a total of 243 within-class plasma FA concentrations across 16 total lipid classes were available for analyses | Yes | Night SBP (associated with change)   \| LPC (22:5) \| <0.05 \| + \| \| --- \| --- \| --- \| |
| **Trimigno, 2023** | Plasma | **INT:** NND  **CON:** ADD | DBP at T2:*  NND: −0.52±0.79 mmHg  ADD: −0.08±0.92 mmHg | 154 variables: 65 were signature signals of known plasma metabolites, 33 unknown spin systems and 56 BINS representing complex regions containing unresolved signals of more than one metabolite | Yes | NND diet  **DBP:**   \| 3-hydroxybutyric acid \| <0.01 \| **-** \| \| --- \| --- \| --- \| \| Acetoacetic acid \| <0.01 \| **-** \| \| Acetone \| <0.01 \| **-** \| \| Succinic acid \| <0.01 \| **-** \| |
| **Changwei, 2023** | Serum | ProBP study | NA | ProBP: 1343 metabolites were quantified,  and 1268 metabolites passed quality control  Significant metabolites identified in the ProBP trial were tested for cross-sectional associations with hypertension in the Bogalusa Heart Study (BHS).  BHS: 1466 metabolites were quantified, and 1202 metabolites passed quality control | Yes | All population  ProBP study  Associated with BP changes  **SBP**   \| Erucate (22:1n9) \| <0.001 \| + \| \| --- \| --- \| --- \|   BHS  Erucate was associated with higher odds of hypertension (odds ratio per unit change = 1.34; 95% confidence interval: 1.07–1.68; *p* = 0.01)  ProBP soy  **SBP**   \| Isobutyrylglycine \| <0.001 \| - \| \| --- \| --- \| --- \| \| Isovalerylglycine \| <0.001 \| - \|   **DBP**   \| Isobutyrylglycine \| <0.001 \| - \| \| --- \| --- \| --- \| \| Isovalerylglycine \| <0.001 \| - \|   ProBP soy and milk  BP   \| Dihomo-linolenoyl-choline \| <0.001 \| - \| \| --- \| --- \| --- \| \| Oleoylcholine \| <0.001 \| - \| |
| **Sun, 2024** | Plasma | ProBP study ProBP-soy, soy protein supplement ProBP-milk, milk protein supplement ProBP-carbs, carbohydrates supplement | Only measured at baseline.  All ProBP study population:  SBP 126.6±8.9 mmHg  DBP 81.3±5.0 mmHg | The ProBP trial identified 18 metabolites associated with BP, including 9 for SBP and 9 for DBP. Except for 1 identified at baseline, 17 metabolites were associated with BP at the end of dietary interventions: 8 from the carbohydrate diet, 4 from the milk protein diet, and 5 from the soy protein diet. | Unclear | Association between metabolites and BP  **SBP**  Carbohydrate Intervention   \| X - 23276 \| <0.001 \| - \| \| --- \| --- \| --- \|   Milk Protein Intervention   \| Glycerol \| <0.001 \| + \| \| --- \| --- \| --- \| \| Hexadecadienoate (16:2n6) \| <0.001 \| + \| \| Alpha-ketobutyrate \| <0.001 \| + \| \| Palmitoyl-arachidonoyl-glycerol (16:0/20:4) [2] \| <0.001* \| + \|   Soy Protein Intervention   \| N-stearoyl-sphinganine (d18:0/18:0) \| <0.001 \| + \| \| --- \| --- \| --- \| \| N6-carbamoylthreonyladenosine \| <0.001* \| + \| \| 5-HEPE \| <0.001 \| + \| \| Alpha-CEHC sulfate \| <0.001 \| + \|   **DBP**  Pretrial baseline   \| 3-indoleglyoxylic acid \| <0.001 \| + \| \| --- \| --- \| --- \|   Carbohydrate Intervention   \| 1-linoleoyl-GPE (18:2) \| <0.001 \| + \| \| --- \| --- \| --- \| \| 1-oleoyl-GPE (18:1) \| <0.001* \| + \| \| 1-palmitoyl-2-oleoyl-GPE (16:0/18:1) \| <0.001 \| + \| \| 1-stearoyl-2-linoleoyl-GPC (18:0/18:2) \| <0.001 \| + \| \| 1-stearoyl-2-linoleoyl-GPE (18:0/18:2) \| <0.001* \| + \| \| 1-stearoyl-2-oleoyl-GPE (18:0/18:1) \| <0.001 \| + \| \| N-acetyltyrosine \| <0.001 \| + \|   Soy Protein Intervention   \| Maltose \| <0.001 \| + \| \| --- \| --- \| --- \|   * Did not pass the significance threshold (p < 6.49E-4) in the full model (adjusted for age, sex, race, education, cigarette smoking, alcohol drinking, BMI, and employment status), but did in the base model (adjusted for age, sex, and race) |

Blood pressure is presented as Mean ± Standard Deviation or * Mean ± SEM (standard error of the mean) and §Mean changes in SBP/DBP from baseline (95% CI). ADD, Average Danish diet. DBP, Diastolic blood pressure. HNLP, high phenolics contents. LHLP, low phenolics contents. NA, not available. NND, New Nordic Diet. SBP, Systolic blood pressure.
